# Pilot study of psilocybin in patients with post-treatment lyme disease

**DOI:** 10.1038/s41598-026-38091-9

**Published:** 2026-02-25

**Authors:** Albert Garcia-Romeu, Gideon P. Naudé, Alison W. Rebman, Sara So, Abigail Yaffe, Ian Geithner, Erica A. Kozero, Ting Yang, Mark J. Soloski, John N. Aucott

**Affiliations:** 1 Center for Psychedelic and Consciousness Research, Department of Psychiatry and Behavioral Sciences, Johns Hopkins School of Medicine, 5510 Nathan Shock Drive, Baltimore, MD 21224 USA; 2 Lyme Disease Research Center, Division of Rheumatology, Department of Medicine, Johns Hopkins School of Medicine, Baltimore, MD 21205 USA

**Keywords:** Lyme disease, Signs and symptoms, Quality of life, Posttreatment lyme disease syndrome, Psilocybin, Psychedelics, Psychedelic therapy, Diseases, Health care, Medical research, Microbiology, Neuroscience

## Abstract

Lyme disease, caused by the bacterium *Borrelia burgdorferi*, is the most common vector-borne disease in the United States and Europe. Although antibiotics effectively treat most cases, an estimated 10–20% of patients develop post-treatment Lyme disease (PTLD), a chronic syndrome marked by fatigue, pain, cognitive difficulties, mood disturbance, and reduced quality of life. There are no established treatments for PTLD. The serotonin 2A receptor agonist psychedelic psilocybin has recently shown potential antidepressant and anxiolytic effects in clinical trials as well as preliminary evidence for anti-inflammatory effects in animals. This open-label, single-arm pilot study evaluated the effects of psilocybin in 20 participants with well-characterized PTLD. The 8-week intervention included two psilocybin sessions (15 mg in week 4; 15 or 25 mg in week 6) with psychological support. Participants (11 women, 9 men, mean age 44, median illness duration 5.7 years) showed significant improvements in PTLD symptom burden and quality of life from study enrollment through 1-month following the second dose of psilocybin (primary endpoint), with significant benefits sustained through 6 months. At the 6-month follow-up, general PTLD symptom burden (GSQ-30) was decreased 40% from baseline (*p* < .001; Cohen’s d_z_ = − 1.22, 95% CI [− 2.12, − 0.78]), and health-related quality of life improved across both SF-36 domains, with Mental (MCS) and Physical (PCS) component scores increasing 13% (*p* ≤ .013; Cohen’s d_z_ = 0.46–0.59). Secondary outcomes assessing mood, fatigue, sleep quality, and pain also showed significant and sustained improvement through 6 months (all *p* ≤ .003; Cohen’s d_z_ = 0.56–1.25). No serious adverse events (AEs) related to the study intervention occurred. The most common AEs attributed to psilocybin were transient hypertension (90%), headache (65%), tachycardia (35%), pain (20%), and fatigue (15%). Preliminary findings support that psilocybin-assisted treatment was feasible and well-tolerated among the sample. Clinical outcomes indicated potentially long-lasting benefits of psilocybin-assisted treatment, warranting further investigation for PTLD.

Trial registration: ClinicalTrials.gov Identifier NCT05305105, registered 22/03/2022.

**Supplementary Information:**

The online version contains supplementary material available at 10.1038/s41598-026-38091-9.

## Introduction

Lyme disease is an inflammatory infectious disease caused by the spirochete bacterium *Borrelia burgdorferi*. Cases of Lyme disease have been increasing steadily over the past several decades, and it is currently the most common vector-borne disease in the United States (U.S.) and Europe, with an estimated 465,000 individuals treated for Lyme disease annually in the US^1^. While early and late disease can often be treated successfully with antibiotic medications, an estimated 10–20% of patients develop chronic symptoms after treatment including fatigue, pain, cognitive difficulties, and sleep disturbance, which has been characterized as post-treatment Lyme disease (PTLD)^2,3^. In addition to physical and cognitive complaints, PTLD patients also struggle with neuropsychiatric symptoms (e.g., anxiety, depression) and diminished quality of life^4^. Available clinical data suggest over 40% of those with PTLD may suffer from major depression^4,5^, and nearly a third meet criteria for an anxiety disorder^5^, with additional evidence indicating elevated levels of affective disorders, suicide attempts, and completed suicide among individuals diagnosed with Lyme disease^6^. While a research case definition for PTLD has been proposed^7^, the condition remains controversial due to lack of validated biomarkers and poorly understood mechanisms. There are currently no accepted treatments for PTLD^8^, and as such, patients often face substantial challenges when seeking diagnosis and treatment^9^.

Recent research suggests the naturally occurring serotonin 2A receptor (5-HT_2A_R) agonist psychedelic psilocybin can produce rapid-acting and enduring antidepressant and anxiolytic effects after a single high dose^10–13^. Psilocybin and other ‘classic psychedelics’ such as lysergic acid diethylamide (LSD) have also shown analgesic and quality of life enhancing effects in patients with life-threatening illnesses, such as cancer^12–17^, as well as preliminary evidence of clinically significant improvements in neurological conditions including migraine and cluster headaches^18–20^. Although the specific mechanisms underlying this diverse array of potential benefits remain unclear, some medicinal effects of classic psychedelics might relate to their anti-inflammatory properties^21,22^. Additionally, several potential neurobiological and psychological mechanisms may contribute to psychedelics’ therapeutic effects, such as altered gene expression^23^, enhanced neural plasticity^24^, large-scale functional connectivity changes^25^, and improvements in cognitive flexibility and psychological insight^26^. Taken together, current evidence indicates that classic psychedelics may hold promise for mitigating symptoms and improving quality of life in patients with treatment-resistant chronic illnesses, such as PTLD. The present open-label pilot study was conducted to examine the impact of psilocybin-assisted treatment with psychological support in a well-characterized cohort of 20 patients with PTLD.

## Methods

This study was approved by an Institutional Review Board at the Johns Hopkins University School of Medicine and was performed in accordance with the 1964 Declaration of Helsinki. Participants provided their written informed consent.

### Participants

The primary source of recruitment for this study was patients who had previously participated in studies at the Johns Hopkins Lyme Disease Research Center and who had given consent to future contact. Additional participants were recruited via online advertisements and word of mouth. Interested individuals underwent screening via online questionnaire and phone screen to assess major inclusion/exclusion criteria before in-person screening.

### Eligibility criteria

To be eligible to participate in this trial, volunteers were required to meet the following criteria: be ≥ 18 years of age; capable of providing written informed consent; willing to allow the study team to review past medical records; and have medical record documentation of meeting the CDC case definition for clear diagnosis and treatment of early or late Lyme disease prior to the onset of post-treatment Lyme disease (PTLD). In line with the Infectious Disease Society of America’s (IDSA) proposed criteria for PTLD^7^, study eligibility required a history of physician-documented single or disseminated erythema migrans rash, late Lyme arthritis, or late Lyme neuropathy, OR medical record documentation of meeting the CDC case definition for probable early or late Lyme disease, including a history of abrupt onset of flu-like symptoms with or without a misdiagnosed rash, and concurrent positive serology, as well as having received treatment with a recommended course of antibiotics, and at least one current PTLD-defining symptom (widespread pain, fatigue, or neurocognitive dysfunction) following completion of standard, recommended antibiotic therapy for treatment of Lyme disease, and that appeared in the first two years following first evidence of Lyme disease. Participants were also required to be medically stable as determined by screening for medical exclusion criteria (see list below) via a personal interview, a medical questionnaire, a physical examination, an electrocardiogram (ECG), and routine medical blood and urinalysis laboratory tests. Consistent with recent findings on safety of co-administration of psilocybin with SSRI medications^27,28^, concurrent use of SSRIs, SNRIs, and/or bupropion were allowed during the trial, given that the type and frequency of the therapy had been stable for at least two months prior to screening. Allowable bupropion dose was ≤ 300 mg/day due to concerns about potential for seizures in combination with psilocybin^29^. There were no upper limits for SSRI and SNRI daily doses.

### Exclusion criteria

Exclusion criteria were assessed via comprehensive medical record review, structured interviews, medical and mental health history questionnaires, and physical examination. Individuals were excluded from the study if they met DSM-5 criteria for moderate or severe substance use disorder (excluding tobacco) within the past 5 years; were currently taking antipsychotics, MAO inhibitors, or antidepressant medications other than SSRIs, SNRIs, or bupropion; were currently taking lithium or other primary centrally-acting serotonergic medications, whether over-the-counter or prescription (e.g., efavirenz, 5-hydroxytryptophan, St. John’s wort); had current or prior history of major immunosuppressive illness or medications; exhibited cardiovascular conditions, including angina, a clinically significant ECG abnormality (e.g. atrial fibrillation or QTc > 450msec), transient ischemic attack (TIA) in the last 6 months, stroke, artificial heart valves, or uncontrolled hypertension with resting blood pressure > 139 systolic or > 89 diastolic, or heart rate > 90 bpm at screening; showed evidence of renal disease (i.e., creatinine clearance < 40 ml/min using the Cockraft and Gault equation); had current or past history of meeting DSM-5 criteria for schizophrenia, psychotic disorder (unless substance-induced or due to a medical condition), or bipolar I or II disorder; had a first degree relative with history of schizophrenia, psychotic disorder (unless substance-induced or due to a medical condition), or bipolar I disorder; reported past-year hallucinogen use; received the Lyme vaccine when it was available (1998–2002); had developed unexplained chronic pain, chronic fatigue syndrome, fibromyalgia, autoimmune disease, or unexplained neurologic symptoms before first evidence of Lyme disease based on medical records and self-report; had cancer or malignancy in the past 2 years; epilepsy with history of seizures; insulin-dependent diabetes; current dementia or related disorders (e.g., Alzheimer’s Disease, vascular dementia, Lewy body dementia, and frontotemporal disorders); were currently pregnant or nursing; were currently of childbearing potential and not using effective methods of contraception; were not fluent in English; or at high risk for suicidal ideation or behavior (i.e., reporting suicidal ideation with intent or behavior on the C-SSRS at screening, or individuals with a suicide attempt within the past 3 years).

### Procedures

Upon enrollment, participants completed an 8-week study intervention including two psilocybin sessions with psychological support, and follow-up assessments 1, 3, and 6 months after the final psilocybin session. The study intervention included standardized psychoeducation about psilocybin effects prior to dosing, a brief life review to discuss the course of illness, symptom burden, and other formative life events (e.g., trauma, key relationships, work history), and participant guided formation of desired treatment goals with two trained study facilitators. Following a person-centered approach, discussion topics and treatment goal formation were primarily driven by participants, their expressed preferences, and their experiences, with minimally scripted didactic content to guide the conversations (see treatment manual in Supplementary Materials). Facilitators included at least one master’s or doctoral level psychologist or psychiatrist and a co-facilitator with at least a bachelor’s degree and one year of experience in a mental health-related field. After 3 weekly preparatory meetings, participants received a single moderate dose (15 mg) of psilocybin in week 4 of the 8-week intervention, and either a moderately high dose (25 mg) or another moderate dose (15 mg) in week 6 depending on intensity of participant response in the first session. After each psilocybin session, participants completed a follow-up integration meeting within 3 days to assess their mental status and document any adverse events. Weekly meetings with facilitators were conducted through week 8 to discuss the contents of participants’ psilocybin session and to monitor their overall progress. Study visits were primarily conducted virtually via secure online video-chat (i.e., Zoom), apart from screening, dosing sessions, and the week 8 and 6-month follow-up visits that were conducted in-person at the Johns Hopkins Bayview Campus. Participants completed post-session assessments at approximately weeks 8 and 10 (i.e., 2 weeks and 1 month after the 2nd psilocybin session, respectively), and again at follow-ups approximately 3- and 6-months after the final psilocybin session. The data presented here were collected between July 1, 2022, and April 7, 2025.

### Adverse events

From the first dosing session through the final follow-up, adverse events (AEs) were assessed by asking participants, “how have you been feeling since your last visit?” Staff recorded any new or worsening physical or mental health events self-reported or observed during study visits. AEs were deemed related or unrelated to the study intervention based on temporal proximity, participant attribution, and investigators’ judgment. Adverse events were monitored through resolution and collated according to major organ and system class using the MedDRA v.28.0 classification system.

### Primary outcomes: symptom burden and quality of life

####  General Symptom Questionnaire-30 (GSQ-30)

 The GSQ-30 is a 30-item instrument developed and validated in collaboration with members of the Johns Hopkins Lyme Disease Research Center^30^. The GSQ-30 is a valid and reliable means to assess multi-system symptom burden among patients with PTLD, including pain, fatigue, neuropsychiatric, neurologic, and viral-like symptoms. Higher GSQ-30 scores indicate greater symptom burden during the past 2 weeks.

####  Short-Form Health Survey (SF-36)

The SF-36 is a multi-purpose, short-form 36-item health survey that yields an 8-scale profile of functional health and well-being, as well as psychometrically based mental and physical health summary scores^31^. It is a valid and reliable generic indicator of health status, with well-established normative data and scoring distributions across clinical and general populations^32^. Higher scores on SF-36 subscales and mental and physical composite summaries indicate better health-related quality of life.

### Secondary outcomes: mood, sleep, fatigue, and pain domains

####  Beck Depression Inventory-II (BDI-II)

The BDI-II is a widely used, 21-item measure of cognitive and vegetative symptoms of depression^33^. It demonstrates good reliability and validity and can be scored to include or exclude somatic symptoms. Total scores on the BDI-II range from 0 to 63, with higher scores signifying greater depression severity. Scores from 14 to 19 indicate mild depression, 20–28 moderate depression, and 29–63 severe depression.

####  Pittsburgh Sleep Quality Index (PSQI)

The PSQI is a 19-item self-report inventory that assesses sleep quality across several domains, including daytime dysfunction, sleep latency, duration, disturbance, quality and efficiency^34^. Total scores on the PSQI may range from 0 to 21, with higher scores indicating worse sleep quality, and scores above 5 suggesting sleep difficulties.

####  Fatigue Severity Scale (FSS)

The FSS was designed to detect and evaluate changes in fatigue over time in people with chronic illness^35^. Its utility in research rests with its ability to measure fatigue severity and distinguish fatigue from other clinical features of chronic medical disorders, such as depression. Total FSS scores can range from 9 to 63, with higher scores indicating greater fatigue severity, and scores above 35 suggesting potential fatigue.

####  Short-Form McGill Pain Questionnaire (SF-MPQ)

 The SF-MPQ provides a quantitative assessment of pain using major classes of word descriptors reported by patients to characterize their subjective pain experience^36^. It includes an 11-item sensory subscale and a 4-item affective subscale, allowing for a multidimensional evaluation of pain experience. SF-MPQ sensory subscale scores may range from 0 to 33, affective subscale scores may range from 0 to 12, and total (summed) scores may range from 0 to 45, with higher subscale and total scores indicating greater pain.

### Statistical analysis

Pre-registered primary and secondary outcome endpoints (described above) assessed changes from baseline scores at 2-weeks and 1-month after the final psilocybin session. Exploratory endpoints examined longer-term changes in outcomes at 3 and 6 months after the final psilocybin session. Changes in primary and secondary outcomes were assessed using separate linear mixed-effects models fitted via restricted maximum likelihood estimation, which was applied consistently across all outcomes. Time was modeled as a five-level categorical fixed effect and a random intercept for participant was included to account for repeated measures. Pairwise comparisons were conducted between baseline (reference time point) and the study visits that took place approximately 2 weeks, 1 month, 3 months, and 6 months after the second psilocybin dosing session. Confidence intervals and p-values for fixed effects were obtained using the Kenward-Roger (KR) approximation.

Within-subject effect sizes were estimated using Cohen’s d_z_ (the standardized mean difference for repeated measures), with 95% confidence intervals constructed using the percentile bootstrap method (2000 resamples). Robustness was assessed by repeating analyses after excluding multivariate outliers based on Mahalanobis distance. Outliers were defined across the five time points using a chi-square cutoff for 5 degrees of freedom (χ^2^ = 11.07, *p* < 0.05; see Supplementary Materials and Supplementary Fig. 1). P-values were adjusted for multiple comparisons using the Benjamini–Hochberg false discovery rate procedure, and all tests were two-sided. Model assumptions, including normality of residuals and homoscedasticity, were assessed via diagnostic plots. Analyses were performed in R (version 4.4.1)^37^ and utilized the *lme4*^38^, *lmerTest*^39^, *performance*^40^, and *boot*^41^ packages.

## Results

### Participant characteristics

**Fig. 1:**
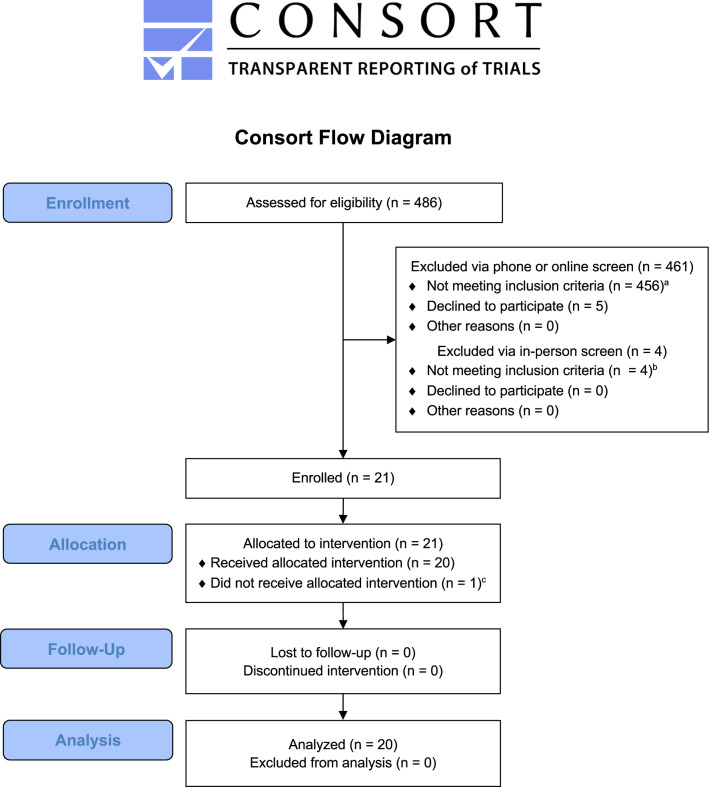
Consort flow diagram. ^a^Disqualified at initial phone/online screening reasons: Cancer or malignancy in the past two years (*n* = 11); Cardiovascular Risks (High BP or Abnormal EKG; *n* = 31); Current dementia (e.g. Alzheimer’s Disease, *n* = 2); Not responsive (*n* = 11); Insulin-dependent diabetes (*n* = 2); Psychiatric comorbidities (e.g., alcohol use disorder, *n* = 7); No medical record documentation of meeting the CDC case definition for clear diagnosis and treatment of Lyme disease (*n* = 87); Development of unexplained chronic pain, chronic fatigue syndrome, fibromyalgia, autoimmune disease, or unexplained neurologic symptoms before first evidence of Lyme disease (*n* = 25); Concerns about time / travel commitment (*n* = 117); Current use of exclusionary medications (e.g. antipsychotics, *n* = 71); Recent or extensive hallucinogen use (*n* = 23); Seizure disorders (*n* = 8); Family history of psychiatric illness (e.g., schizophrenia, *n* = 14); High risk for suicidal ideation (*n* = 16); Renal disease (*n* = 1); Did not receive recommended course of antibiotics when initially diagnosed with Lyme (*n* = 23); Received the Lyme vaccine when it was available (1998–2002) (*n* = 7). ^b^Disqualified at in-person screening reasons: High risk for suicidal ideation or behavior (*n* = 1); Current dementia or related disorder (*n* = 1); Family history of bipolar disorder (*n* = 1); Not medically stable as determined at screening (*n* = 1). ^c^Declined to participate in psychological support component of trial: *n* = 1.

Figure 1 summarizes trial recruitment and retention. In total, 486 individuals were pre-screened, of whom 461 were disqualified for not meeting criteria (*n* = 456) or declining participation (*n* = 5). The most common pre-screening exclusionary factors were related to failing to meet our full PTLD criteria (*n* = 142; 30.9%), concerns about travel or time, or non-responsive (*n* = 128; 27.8%), exclusionary co-morbidities (*n* = 96; 20.9%), and current exclusionary medication or recent hallucinogen use (*n* = 94; 20.4%). Twenty-five individuals were consented and screened in-person, of whom 4 were disqualified for not meeting study criteria. One was enrolled but dropped out before receiving psilocybin due to unwillingness to engage in the psychological support portion of the intervention, resulting in a final sample of *N* = 20 enrolled and receiving the study intervention (i.e., 4.1% of those pre-screened).

Two participants (10.0%) received 15 mg psilocybin in both dosing sessions due to meeting a priori response criteria (i.e., ≥ 60% of the maximum total score on the Challenging Experience Questionnaire^42^ after the first session, with the remainder (*n* = 18; 90.0%) receiving 15 mg followed by 25 mg psilocybin separated by approximately two weeks per protocol. Participant demographics are shown in Table 1. The sample had a mean age of 44.05 (SD = 10.10), 11 (55.0%) were female, and the majority identified as white (90.0%). The median duration of illness from Lyme disease onset to study screening was 5.74 years (range = 2.06–21.78). Initial Lyme presentations were most commonly viral-like illness and positive test only (60.0%), followed by late Lyme arthritis (20.0%), neurologic disease (10.0%), and erythema migrans only (10.0%). At screening, five participants met criteria for major depression (25.0%), five met criteria for attention deficit-hyperactivity disorder (ADHD; 25.0%), and one additionally met criteria for panic disorder and post-traumatic stress disorder (PTSD; 5.0%). Three participants (15.0%) remained on antidepressants throughout the trial, five were on ADHD medications (25.0%), and four (20.0%) reported previous use of hallucinogens.

**Table 1:** Participant demographics, (*N* = 20). ^a^One patient with neurologic disease and one patient with late Lyme arthritis also had an erythema Migrans rash; ^b^for neurologic presentation, one participant reported bell’s palsy and another reported postural orthostatic tachycardia syndrome (POTS); BMI, Body mass index; ADHD, Attention-deficit/hyperactivity disorder, PTSD, Post-Traumatic stress Disorder.

Age, m (SD)	44.1 (10.1)
Sex, *n* (% female)	11 (55.0)
Weight (lb), *m* (*SD*)	180.6 (49.6)
BMI (kg/m^2^), *m* (*SD*)	27.9 (6.7)
Race, *n* (%)	
White	18 (90.0)
South Asian	2 (10.0)
Education, *n* (%)	
High school diploma	1 (5.0)
Some college	1 (5.0)
Associates	1 (5.0)
Bachelors	10 (50.0)
Masters	5 (25.0)
Doctoral	2 (10.0)
State of residence	
Maryland	6 (30.0)
Pennsylvania	6 (30.0)
Virginia	4 (20.0)
District of Columbia	1 (5.0)
New Jersey	1 (5.0)
Connecticut	1 (5.0)
North Carolina	1 (5.0)
Illness duration (years) from Lyme onset to study screening, *mdn* (range)	5.7 (2.1–21.8)
Initial Lyme clinical presentation, *n* (%)	
Erythema migrans only	2 (10.0)
Neurologic disease^a,b^	2 (10.0)
Late Lyme arthritis^a^	4 (20.0)
Viral-like illness and positive serology	12 (60.0)
Comorbid psychiatric diagnoses at screening	
ADHD	5 (25.0)
Major depression	5 (25.0)
Panic disorder	1 (5.0)
PTSD	1 (5.0)
Previous hallucinogen use, *n* (%)	4 (20.0)

### Adverse events

No serious adverse events (AEs) related to the study intervention occurred. All 20 participants reported an AE during the study period, and all 20 experienced AEs related to the intervention either during or immediately after the dosing sessions. The most common AEs attributed to psilocybin were hypertension (90%), headache (65%), tachycardia (35%), pain (e.g., in back, neck, or extremities; 20%), and fatigue (15%). See Table 2 for a summary of common AEs and Supplementary Tables 1 and 2 for a full listing. One participant experienced a serious adverse event during the study period involving passive suicidal ideation (SI) after initiating a new antidepressant pharmacotherapy. This occurred approximately seven weeks after their final exposure to psilocybin and is a known side effect of the medication they initiated. SI resolved within four days of stopping the antidepressant medication under their prescribing physician’s direction and was deemed unrelated to the psilocybin-assisted treatment. Another participant reported developing Stage 3 rectal cancer at their 6-month follow-up visit that was deemed unrelated to the psilocybin-assisted treatment.

**Table 2:** Summary of adverse events during the 6-week trial period and on session days. ^a^For the purposes of adverse event (AE) reporting, the 6-week trial period refers to the time from the first psilocybin administration in week 4 of the intervention through the 1-month follow-up in approximately week 10. for a full listing of AEs throughout the trial see supplementary tables 1 and 2. ^b^The protocol called for the second session to be at 15 mg or 25 mg depending on response to the first session. In this study 2 participants received a 15 mg dose in session 2, while the remaining 18 received 25 mg. ^c^Relationship of adverse event to the therapeutic intervention was determined by the study team based on Temporal proximity, participant attribution, and clinical judgment. Events deemed “probably” or “definitely” related were counted here. ^d^One participant with depression reported passive suicidal ideation after starting a new serotonin-norepinephrine reuptake inhibitor (SNRI) antidepressant approximately 7 weeks after their final psilocybin session. they stopped the medication under direction of the prescribing physician and this resolved within 4 days.

Adverse events	6-Weektrial period^a^		Session Day 1 (15 mg)	Session Day 2 (25 mg)^b^
	Incidence	Related^c^	Incidence	Incidence
Any adverse event, n (%)	20 (100%)	20 (100%)	14 (70%)	18 (90%)
Serious adverse event	1 (5%) ^c^	0 (0%)	0 (0%)	0 (0%)
Adverse event reported in > = 2 participants during the trial				
Hypertension	18 (90%)	18 (90%)	12 (60%)	16 (80%)
Headache	14 (70%)	13 (65%)	6 (30%)	8 (40%)
Tachycardia	7 (35%)	7 (35%)	1 (5%)	6 (30%)
Pain	6 (30%)	4 (20%)	2 (10%)	2 (10%)
Fatigue	4 (20%)	3 (15%)	1 (5%)	1 (5%)
Anxiety	3 (15%)	0 (0%)	0 (0%)	0 (0%)
COVID-19	3 (15%)	0 (0%)	0 (0%)	0 (0%)
Low mood	3 (15%)	1 (5%)	0 (0%)	1 (5%)
Depression ^d^	2 (10%)	0 (0%)	0 (0%)	0 (0%)
Insomnia	2 (10%)	0 (0%)	0 (0%)	1 (5%)
Visual disturbance	2 (10%)	0 (0%)	0 (0%)	0 (0%)
Vomiting	2 (10%)	1 (5%)	0 (0%)	1 (5%)

Blood pressure and heart rate were regularly monitored during psilocybin sessions. We observed modest elevations in both blood pressure and heart rate that were anticipated and resolved without intervention. Other than use of over-the-counter treatments for headache or pain, no other interventions were required for managing study-related adverse events. Peak vital signs during the first and second psilocybin sessions were: mean (SD) systolic blood pressure = 142.10 (9.79) and 143.65 (11.40) mmHg; diastolic blood pressure = 89.00 (9.21) and 91.25 (6.17) mmHg; and heart rate = 81.05 (11.53) and 89.70 (17.05) bpm, respectively. Peak heart rate was modestly higher during the second session, though all values remained within typical ranges observed in psilocybin studies.

### Outcome measures

**Fig. 2:**
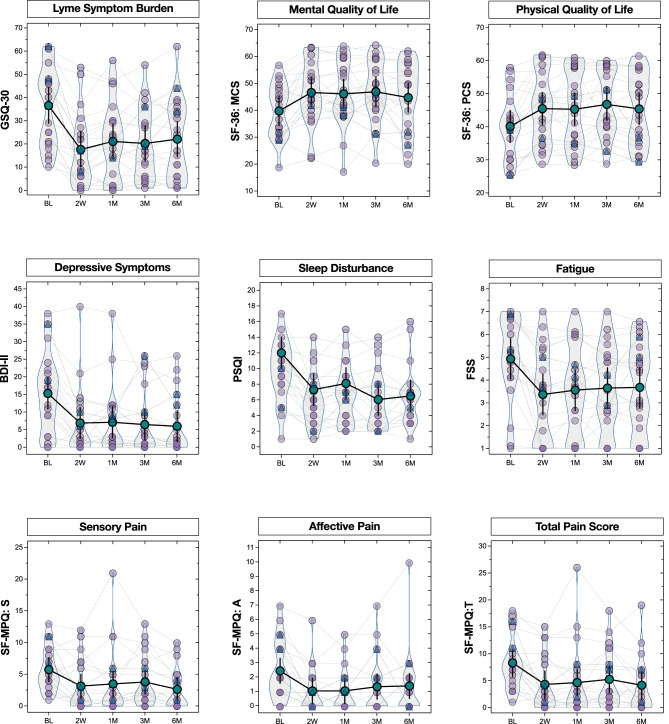
Primary and secondary outcomes across baseline and follow-up timepoints. Plots display individual scores, distributions, and summary statistics at baseline, 2 weeks, 1 month, 3 months, and 6 months after the second psilocybin session. Larger circles represent the estimated sample mean, with vertical bars indicating 95% confidence intervals derived from the linear mixed-effects model (Kenward–Roger). Triangles represent participants who repeated the 15 mg dose (*n* = 2). All pairwise comparisons differed significantly from baseline at FDR-adjusted *p* ≤ .013. Row 1 (primary outcomes): General Symptom Questionnaire-30 (GSQ-30; higher scores indicate greater general symptom burden), SF-36 Mental Component Summary, and SF-36 Physical Component Summary scores (higher scores reflect better mental and physical quality of life, respectively). Row 2 (secondary outcomes): Beck Depression Inventory-II (BDI-II; higher scores indicate greater depressive symptoms), Pittsburgh Sleep Quality Index (PSQI; higher scores indicate greater sleep disturbance), and Fatigue Severity Scale (FSS; higher scores indicate greater fatigue). Row 3 (secondary outcomes): Short-Form McGill Pain Questionnaire (SF-MPQ) Sensory and Affective subscales and Total score (higher scores indicate greater pain).

Linear mixed-effects models revealed a significant main effect of time for all nine outcomes (all F(4, 76) ≥ 4.56, all *p* ≤ .002). Figure 2 depicts individual trajectories and model-estimated means, with distinct symbols representing participants who repeated the 15 mg dose (*n* = 2). Table 3 presents pairwise contrasts. Baseline model intercepts are provided in Supplementary Table 3.1–3. The study’s primary outcome, general symptom burden (GSQ-30), decreased by 51.7% at 2 weeks (b = − 18.90, 95% CI [− 23.56, − 14.24]) and by 42.3% at 1 month (b = − 15.45, 95% CI [− 20.11, − 10.79]), remaining approximately 40–50% below baseline through 6 months after the second psilocybin dose (F(4, 76) = 20.37, *p* < .001). Quality of life also improved significantly following treatment. Scores on the SF-36 Mental Component Summary (MCS) increased by 17.2% at 2 weeks (b = 6.85, 95% CI [2.96, 10.75]) and 16.0% at 1 month (b = 6.37, 95% CI [2.47, 10.26]), remaining approximately 12–18% above baseline through 6 months (F(4, 76) = 4.56, *p* = .002). Scores on the SF-36 Physical Component Summary (PCS) increased by 13.4% at 2 weeks (b = 5.38, 95% CI [2.14, 8.62]) and 12.9% at 1 month (b = 5.17, 95% CI [1.93, 8.41]), remaining approximately 13–17% higher through 6 months (F(4, 76) = 5.07, *p* = .001).

**Table 3:** Estimated changes in clinical outcomes at 2 weeks, 1 month, 3 months, and 6 months after final psilocybin administration.

Outcome	Time	Estimate (95% CI)^a^	% Δ (Baseline)	*P*-value^b^	d_z_ (95% CI)^c^
GSQ-30	2 wk	− 18.90 (− 23.56, − 14.24)	− 51.7%	< 0.001	− 1.51 (− 2.12, − 1.20)
	1 mo	− 15.45 (− 20.11, − 10.79)	− 42.3%	< 0.001	− 1.12 (− 1.71, − 0.80)
	3 mo	− 16.35 (− 21.01, − 11.69)	− 44.7%	< 0.001	− 1.40 (− 2.08, − 1.04)
	6 mo	− 14.50 (− 19.16, − 9.84)	− 39.7%	< 0.001	− 1.22 (− 2.12, − 0.78)
SF-36					
Mental summary score	2 wk	6.85 (2.96, 10.75)	17.2%	< 0.001	0.69 (0.32, 1.17)
	1 mo	6.37 (2.47, 10.26)	16.0%	0.002	0.64 (0.24, 1.15)
	3 mo	7.14 (3.24, 11.03)	18.0%	< 0.001	0.73 (0.37, 1.18)
	6 mo	4.98 (1.08, 8.87)	12.5%	0.013	0.46 (0.05, 0.93)
Physical summary score	2 wk	5.38 (2.14, 8.62)	13.4%	0.002	0.85 (0.59, 1.26)
	1 mo	5.17 (1.93, 8.41)	12.9%	0.002	0.59 (0.13, 1.25)
	3 mo	6.67 (3.43, 9.90)	16.6%	< 0.001	0.82 (0.32, 1.67)
	6 mo	5.29 (2.06, 8.53)	13.2%	0.002	0.59 (0.15, 1.28)
BDI-II	2 wk	− 8.40 (− 11.48, − 5.32)	− 54.9%	< 0.001	− 1.13 (− 1.87, − 0.72)
	1 mo	− 8.20 (− 11.28, − 5.12)	− 53.6%	< 0.001	− 1.01 (− 1.55, − 0.72)
	3 mo	− 8.85 (− 11.93, − 5.77)	− 57.8%	< 0.001	− 1.02 (− 1.64, − 0.62)
	6 mo	− 9.35 (− 12.43, − 6.27)	− 61.1%	< 0.001	− 1.25 (− 1.82, − 0.91)
PSQI	2 wk	− 4.70 (− 6.19, − 3.21)	− 39.2%	< 0.001	− 1.11 (− 1.57, − 0.82)
	1 mo	− 3.90 (− 5.39, − 2.41)	− 32.5%	< 0.001	− 0.92 (− 1.35, − 0.65)
	3 mo	− 5.95 (− 7.44, − 4.46)	− 49.6%	< 0.001	− 1.18 (− 1.68, − 0.87)
	6 mo	− 5.50 (− 6.99, − 4.01)	− 45.8%	< 0.001	− 1.17 (− 1.73, − 0.85)
FSS	2 wk	− 1.57 (− 2.13, − 1.00)	− 31.8%	< 0.001	− 0.93 (− 1.32, − 0.71)
	1 mo	− 1.37 (− 1.94, − 0.81)	− 27.8%	< 0.001	− 0.76 (− 1.16, − 0.44)
	3 mo	− 1.28 (− 1.85, − 0.72)	− 26.0%	< 0.001	− 0.83 (− 1.44, − 0.42)
	6 mo	− 1.26 (− 1.82, − 0.69)	− 25.5%	< 0.001	− 0.84 (− 1.37, − 0.46)
SF-MPQ					
Sensory pain score	2 wk	− 2.60 (− 3.85, − 1.35)	− 44.8%	< 0.001	− 0.83 (− 1.48, − 0.43)
	1 mo	− 2.25 (− 3.50, − 1.00)	− 38.8%	< 0.001	− 0.57 (− 1.30, − 0.12)
	3 mo	− 1.95 (− 3.20, − 0.70)	− 33.6%	0.003	− 0.65 (− 1.26, − 0.28)
	6 mo	− 3.10 (− 4.35, − 1.85)	− 53.4%	< 0.001	− 1.04 (− 1.69, − 0.65)
Affective pain score	2 wk	− 1.40 (− 2.04, − 0.76)	− 56.0%	< 0.001	− 0.87 (− 1.30, − 0.58)
	1 mo	− 1.40 (− 2.04, − 0.76)	− 56.0%	< 0.001	− 0.91 (− 1.46, − 0.54) ^d^
	3 mo	− 1.10 (− 1.74, − 0.46)	− 44.0%	0.001	− 0.80 (− 1.23, − 0.48)
	6 mo	− 1.05 (− 1.69, − 0.41)	− 42.0%	0.002	− 0.56 (− 1.08, − 0.16)
Total pain score	2 wk	− 4.00 (− 5.58, − 2.42)	− 48.2%	< 0.001	− 0.99 (− 1.67, − 0.64)
	1 mo	− 3.65 (− 5.23, − 2.07)	− 44.0%	< 0.001	− 0.78 (− 1.71, − 0.30)
	3 mo	− 3.05 (− 4.63, − 1.47)	− 36.7%	< 0.001	− 0.92 (− 1.67, − 0.48)
	6 mo	− 4.15 (− 5.73, − 2.57)	− 50.0%	< 0.001	− 1.00 (− 1.76, − 0.63)

Values reflect estimated mean change from baseline derived from linear mixed-effects models fitted by restricted maximum likelihood (REML) with a random intercept for participant. Time (baseline, 2 wk, 1 mo, 3 mo, 6 mo) was modeled as a categorical fixed effect.

^a^95% confidence intervals (CIs) for fixed effects were obtained using the Kenward–Roger approximation for denominator degrees of freedom.

^b^P-values were adjusted using the Benjamini–Hochberg false discovery rate procedure across all baseline–follow-up contrasts (36 total).

^c^Cohen’s d_z_ = standardized within-subject effect size with percentile-bootstrap 95% CIs (2000 resamples).

^d^The model-estimated mean change and confidence interval appear the same at both time points due to rounding; Cohen’s d_z_ is based on within-participant change scores and therefore differs due to variability.

Higher SF-36 scores indicate improved health-related quality of life, whereas lower scores indicate improvement for all other measures.

GSQ-30, General Symptom Questionnaire-30; SF-36, 36-Item Short-Form Health Survey; BDI-II,  Beck Depression Inventory-II; PSQI, Pittsburgh Sleep Quality Index; FSS, Fatigue Severity Scale; SF-MPQ, Short-Form McGill Pain Questionnaire.

Secondary outcomes assessing mood, sleep, fatigue, and pain also showed significant and durable improvements relative to baseline (Table 3). Depressive symptoms (BDI-II) were approximately 50–60% lower at all follow-up points (F(4, 76) = 12.87, *p* < .001). Sleep disturbance (PSQI) scores were approximately 40–50% lower across follow-ups (F(4, 76) = 20.13, *p *< .001). Fatigue severity (FSS) declined approximately 25–30% across follow-ups (F(4, 76) = 9.74, *p* < .001). Pain scores from the Short-Form McGill Pain Questionnaire (SF-MPQ) also improved. Sensory pain decreased approximately 35–55% across follow-ups (F(4, 76) = 7.14, *p* < .001), affective pain decreased approximately 40–55% (F(4, 76) = 6.54, *p* < .001), and total pain decreased approximately 40–50% (F(4, 76) = 9.34, *p* < .001; see Table 3 note regarding variability). Across all outcomes, within-participant standardized effect sizes were large (d_z_ ≈ 0.6–1.5) and remained stable when excluding multivariate outliers.

## Discussion

This pilot study is the first to systematically investigate the effects of moderate and high-dose psilocybin-assisted treatment in patients with PTLD. A recent case report described improvements in neuropsychiatric Lyme disease symptoms in a 70-year-old male patient self-administering low-dose psilocybin^43^. However, to our knowledge, medically supervised administration of high-dose psilocybin-assisted treatment to patients with well-characterized PTLD has not previously been undertaken in a clinical trial setting. The results of the current trial are preliminary and should be considered with caution. Yet findings underscore the compelling possibility that psilocybin-assisted treatment may significantly mitigate key symptoms of PTLD across several physical and neuropsychiatric domains, that participant quality of life may improve substantially after psilocybin-assisted treatment, and that these benefits can persist well beyond the time course of acute drug effects for some patients.

A growing body of evidence has accumulated over the past decade on the effects of psilocybin, primarily surrounding mental health and psychiatric conditions. Findings have largely converged to support rapid-acting antidepressant effects of a single high-dose of psilocybin lasting from 3 weeks to 6 months or longer^10,11,13^. Additional research shows anxiolytic and quality of life enhancing effects of psilocybin-assisted treatment in patients with serious illness^12,15,44^. From a mechanistic standpoint, data suggest factors such as cognitive flexibility and psychological insight may be positively impacted by high-dose psychedelics^26,45,46^, which may in turn promote therapeutic changes in mood and anxiety related symptoms by disrupting entrenched, maladaptive cognitive and emotional patterns. Subjective effects of classic psychedelics, characterized as ego-dissolving, mystical (i.e., marked by a sense of unity), or emotional breakthroughs, have shown associations with antidepressant and other therapeutic outcomes post-treatment^12,13,47–50^, indicating that the experiential component of these treatments may play a salient role in their persisting effects, or possibly serve as a phenomenological marker of longer-term therapeutic response. Participants in the current trial showed reductions in depressive symptoms on average, consistent with the available literature. Additional subjective effects and qualitative interview data will be analyzed and published separately to inform these aspects of psilocybin-assisted treatment in PTLD.

Although the preponderance of clinical research on psilocybin and other classic psychedelics has focused on psychiatric and mental health conditions, an intriguing offshoot of both historic and contemporary investigation suggests classic psychedelics may hold substantial therapeutic potential for conditions considered more squarely physiological in nature, including pain and headache disorders. Similarly, recent pilot studies have found potential benefits of psilocybin-assisted treatment in conditions such as fibromyalgia^51^ and Parkinson’s Disease^52^. While the lines of demarcation between mind and body are far less well-defined than a simple duality, the biological impact of classic psychedelics has emerged as an area of interest regarding their therapeutic effects. Of potential relevance to psychedelics’ benefits for pain and headache disorders, notable biological effects have been observed during and after classic psychedelic administration in the brain across multiple levels^53–55^. Preclinical evidence has found psychedelics may lead to altered gene expression^23,56,57^ and enhanced structural plasticity^24,58^ and metaplasticity^59^ that could provide other bases for their persisting effects. Results from human neuroimaging studies have demonstrated large-scale changes in brain network functional connectivity and electrophysiological activity both acutely and post-acutely^25,60–64^, providing yet other avenues by which these substances may exert long-term therapeutic effects. Although the present study did not collect neuroimaging data, follow-up research should investigate this area further, particularly considering that the available literature suggests PTLD patients can exhibit significant alterations in brain structure and function compared to healthy controls^65^.

Additionally, classic psychedelics have exhibited potent anti-inflammatory effects in preclinical models, which result in modulation of cytokine signaling and immune function in complex ways that could directly impact conditions like PTLD^66–70^. Inflammatory dysregulation has long been implicated in Lyme disease and related conditions such as PTLD^71–73^. Patients with PTLD have shown increased markers of brain inflammation relative to healthy controls including activated microglia^74^ and increased kynurenine levels^75^, which theoretically may be attenuated by psilocybin based on anti-inflammatory properties of classic psychedelics shown in preclinical studies^21,22^. Although the precise pathophysiology of PTLD and therapeutic mechanisms of psilocybin-assisted treatment still lack conclusive accounts, we hope this investigation will provide useful inroads in illuminating both and encourage further examination of psilocybin and other psychedelics for chronic inflammatory and autoimmune conditions. In the present study, biological samples were collected pre- and post-treatment that will be analyzed and published separately to elucidate potential biomarkers and associated treatment response.

A standing debate in the field of psychedelic-assisted treatment surrounds the role and necessity of psychological support or psychotherapy in mediating treatment response^76,77^. In the current study, we used a model of basic psychological support consistent with that used in prior trials at our laboratory in healthy volunteers and patients with cancer-related existential distress^13,78,79^. This model primarily involves nondirective, person-centered support consisting of psychoeducation on the effects of psilocybin, a brief life review covering health history and current symptom burden, formation of treatment goals, and aftercare to integrate and make meaning of the experience in the treatment process. This approach is designed to facilitate the safe conduct of psilocybin administration without explicit, formal psychotherapy, and for the current trial, seemed both appropriate and effective in the PTLD population. No serious study-related adverse events occurred during the trial and all participants returned for both dosing sessions per protocol, though two (10%) maintained the lower 15 mg dose for both sessions due to strong drug effects in the initial session. The lack of attrition in our study speaks to the feasibility of psilocybin-assisted treatment for patients with PTLD.

Converging data show that administration of psilocybin to screened and prepared participants in controlled settings is relatively safe, with transient headache, nausea, dizziness, anxiety, and hypertension being among the most common adverse events^80^. In the present study, we found a similar profile of anticipated but manageable AEs, including headache and hypertension, and these did not require any intervention outside over-the-counter headache medicine. Thus, results suggest psilocybin would not present any greater risk for patients with PTLD than those with other health conditions given the safeguards applied here. However, more research in larger samples is needed to confirm these preliminary safety data, and careful consideration of AE data collection methods are warranted to provide the most comprehensive information^81^.

The current study had several notable limitations. The lack of blinding, randomization, and control conditions may have contributed to inflation of within-subject effect size estimates and make it impossible to conclude that observed symptom reductions were directly attributable to psilocybin-assisted treatment. Repeated self-report measurement is subject to natural variability, such that extreme baseline scores may partially regress toward typical levels over time independent of treatment effects. Expectancy, which was not assessed, and demand characteristics inherent to an open-label intervention may further amplify perceived and reported symptom change, increasing the apparent magnitude of within-subject effects. To date, the only psychedelic clinical trial to report effects of treatment expectancy found that expectancy was associated with antidepressant response to escitalopram (a comparator treatment), but not psilocybin^82^. However, future studies should systematically assess expectancy to further examine its potential impact in psilocybin-assisted therapy and other treatments.

The use of two psilocybin doses with psychological support as an integrated intervention also limits our ability to surmise the relative contribution of either psilocybin dose alone or the role of psychological support in mediating treatment outcomes. The rationale for the two-dose protocol was largely based on clinical experience of the investigators as well as previous trial data indicating substantial variability of response to psilocybin between individuals that was not accounted for by straightforward biological factors such as body weight or body mass index^83^, and superiority of ascending dose sequences in supporting overall improvements in well-being^78^. Thus, by using a lower 15 mg dose first, we were able to identify individuals who may not tolerate the high 25 mg dose well, and allow them to maintain the lower dose, while safely escalating the dose in the majority of participants to allow them to experience a broader spectrum of psilocybin effects, consistent with evidence that therapeutic efficacy is dose-dependent, with higher doses showing greater antidepressant effects^10^.

As previously noted, the diagnosis of PTLD is made clinically, with no currently approved tests to confirm the diagnosis, similar to other infection-associated chronic illnesses such as long-COVID. Instead, a detailed history demonstrating prior Lyme disease, the presence of current symptoms, and the exclusion of other conditions causing similar symptoms is necessary to increase specificity, as we did with the current study’s patient sample. Therefore, we were limited in determining the precise etiology of participants’ symptoms but relied on stringent exclusion criteria, best practices in accordance with the current state of the science and expert guidelines. Additionally, the sample included participants with comorbid psychiatric diagnoses, including major depression and ADHD, as well as concurrent use of psychotropic medications, which may have impacted study outcomes. We judged inclusion of these individuals to enhance clinical generalizability considering the relative prevalence of these comorbidities and medications in patients with PTLD. However, this heterogeneity complicates interpretation of observed improvements. Another limitation was the sociodemographic homogeneity of the study sample, which was primarily comprised of white adults with relatively high levels of education. While this is a trend that prevails in clinical psychedelic research broadly^71^, this may also reflect the over-representation of white individuals in the overall prevalence of Lyme disease^84^. Finally, cognitive difficulty is considered one of the defining features of PTLD, though data on cognitive functioning pre- and post-treatment were not collected, a limitation that should be addressed in future research.

## Conclusions

This study found that psilocybin-assisted treatment was safe and well-tolerated among a cohort of patients with PTLD, and that on average, participants showed significant improvements post-treatment in general symptom burden and quality of life that typically persisted up to 6 months after the final dose of psilocybin. Biomarkers of treatment response, as well as participants’ firsthand accounts of their experiences in the study treatment will be examined in forthcoming research. We consider the current findings to be sufficiently positive to encourage additional randomized controlled research with psilocybin in patients with PTLD, and further characterization of biological mechanisms using methods such as neuroimaging. PTLD is a condition that is growing in prevalence, that results in substantial detriment to patient quality of life and is currently lacking accepted treatments. Thus, we propose further rigorous investigation of psilocybin and other classic psychedelics as potential avenues for novel, effective treatments for this debilitating condition as well as other infection associated chronic conditions such as Myalgic Encephalomyelitis / Chronic Fatigue Syndrome (ME/CFS), Post-acute sequelae of SARS-CoV-2 infection (PASC), and fibromyalgia, that may benefit from similar approaches.

## Supplementary Information

Below is the link to the electronic supplementary material.

Supplementary Material 1

Supplementary Material 2

## Data Availability

The datasets generated and/or analysed during the current study are not publicly available due to privacy and confidentiality and ongoing analyses. De-identified data from the present study may be made available to scientists with a predefined research analytic and data safeguarding plan that complies with Johns Hopkins University guidelines. Please contact A. Garcia-Romeu at AGarci33@jhmi.edu with data-sharing requests. The R scripts used for statistical analyses and figure generation may be made available to qualified researchers with a predefined analytic plan, upon reasonable request to the corresponding author. Please contact G. Naudé at GNaude1@jhmi.edu or A. Garcia-Romeu at AGarci33@jhmi.edu with code-sharing requests.
