# Supplementary material for: Pilot study of psilocybin in patients with post-treatment lyme disease
Source: Sci Rep. 2026 Feb 25;16:7497. doi: 10.1038/s41598-026-38091-9 (PMC12936178; doi:10.1038/s41598-026-38091-9)
Supplement: Supplementary file 1 — Supplementary Material 1 [file 41598_2026_38091_MOESM1_ESM.docx]

**Supplementary Information**

1. **Supplementary Table 1.** **Adverse Events (AEs) occurring during the 6-week trial period, including on psilocybin session days 1 and 2.**

Supplementary Table 1 lists frequency and percentage of participants experiencing adverse events for all adverse events occurring during the 6-week study period following the first administration of psilocybin. Adverse events are classified by MedDRA Preferred Term (PT) and System Organ Class (SOC). Incidence of AEs that occurred on session days are noted here. Incidence of AEs deemed definitely or probably related to psilocybin is also noted here.

1. **Supplementary Table 2.** **Adverse Events (AEs) reported during the 3 to 6-month follow-up period.**

Supplementary Table 2 lists frequency and percentage of participants experiencing adverse events for all adverse events reported during the 3 and 6-month follow-up visits following the end of the 6-week study treatment period. Adverse events are classified by MedDRA Preferred Term (PT) and System Organ Class (SOC). Incidence of AEs that were reported at each long-term follow-up are noted here. Incidence of AEs deemed definitely or probably related to psilocybin is also noted here.

1. **Supplementary Table 3. Estimated changes in clinical outcomes at 2 weeks, 1 month, 3 months, and 6 months after multivariate outlier removal.**

Estimated mean differences, 95% confidence intervals (CIs), percent change from baseline, FDR-adjusted p-values, and within-participant effect sizes (d_z_) for all clinical outcomes.

1. **Supplementary Tables 3.1–3.3. Linear mixed-effects model results for clinical outcomes: Fixed- and random-effect estimates from full-sample and outlier-excluded sensitivity analyses**

**3.1. General Lyme Disease symptom burden and quality of life:**

- GSQ-30
- SF-36 Mental Summary Score (MCS)
- SF-36 Physical Summary Score (PCS; outliers identified; n = 1)

**3.2. Depressive symptoms, sleep quality, and fatigue:**

- BDI-II (outliers identified; n = 3)
- PSQI
- FSS (outliers identified; n = 1)

**3.3. Sensory, affective, and total pain scores:**

- SF-MPQ Sensory Pain (outliers identified; n = 2)
- SF-MPQ Affective Pain (outliers identified; n = 3)
- SF-MPQ Total Pain Score (outliers identified; n = 2)

1. **Supplementary Figure. Primary and secondary outcomes across baseline and follow-up time points after multivariate outlier removal**

Violin plots with aligned individual data points, participant-level trajectories, and estimated marginal means with 95% confidence intervals.

| **Supplementary Table 1.** **Adverse Events (AEs) occurring during the 6-week trial period, including on psilocybin session days 1 and 2.** | | | | | |  |
| --- | --- | --- | --- | --- | --- | --- |
| **Adverse Event MedDRA Preferred Term (PT)** | **System Organ Class (SOC)** | **6-week trial period ^a^** | | **Incidence on Session 1**  **15 mg, n (%)** | **Incidence on Session 2 ^c^ 25 mg, n (%)** |  |
|  |  | **Incidence,**  **n (%)** | **Related, ^b^ n (%)** |  |  |  |
| Hypertension | Vascular disorders | 18 (90%) | 18 (90%) | 12 (60%) | 16 (80%) |  |
| Headache | Nervous system disorders | 14 (70%) | 13 (65%) | 6 (30%) | 8 (40%) |  |
| Tachycardia | Cardiac disorders | 7 (35%) | 7 (35%) | 1 (5%) | 6 (30%) |  |
| Pain ^d^ | General disorders and administration site conditions | 6 (30%) | 4 (20%) | 2 (10%) | 2 (10%) |  |
| Fatigue | General disorders and administration site conditions | 4 (20%) | 3 (15%) | 1 (5%) | 1 (5%) |  |
| Anxiety | Psychiatric disorders | 3 (15%) | 0 (0%) | 0 (0%) | 0 (0%) |  |
| COVID-19 | Infections and infestations | 3 (15%) | 0 (0%) | 0 (0%) | 0 (0%) |  |
| Depressed mood | Psychiatric disorders | 3 (15%) | 1 (5%) | 0 (0%) | 1 (5%) |  |
| Depression | Psychiatric disorders | 2 (10%) | 0 (0%) | 0 (0%) | 0 (0%) |  |
| Insomnia | Psychiatric disorders | 2 (10%) | 0 (0%) | 0 (0%) | 1 (5%) |  |
| Visual distortion | Nervous system disorders | 2 (10%) | 0 (0%) | 0 (0%) | 0 (0%) |  |
| Vomiting | Gastrointestinal disorders | 2 (10%) | 1 (5%) | 0 (0%) | 1 (5%) |  |
| Abdominal pain | Gastrointestinal disorders | 1 (5%) | 1 (5%) | 1 (5%) | 0 (0%) |  |
| Bradycardia | Cardiac disorders | 1 (5%) | 0 (0%) | 1 (5%) | 1 (5%) |  |
| Carcinoma | Neoplasms benign, malignant and unspecified | 1 (5%) | 0 (0%) | 0 (0%) | 0 (0%) |  |
| Chest pain | Cardiac disorders | 1 (5%) | 0 (0%) | 0 (0%) | 0 (0%) |  |
| Dehydration | Metabolism and nutrition disorders | 1 (5%) | 1 (5%) | 0 (0%) | 1 (5%) |  |
| Dizziness | Nervous system disorders | 1 (5%) | 0 (0%) | 0 (0%) | 0 (0%) |  |
| Emotional distress | Psychiatric disorders | 1 (5%) | 1 (5%) | 1 (5%) | 0 (0%) |  |
| Euphoric mood | Psychiatric disorders | 1 (5%) | 0 (0%) | 0 (0%) | 0 (0%) |  |
| Eye infection | Infections and infestations | 1 (5%) | 0 (0%) | 0 (0%) | 0 (0%) |  |
| Herpes labialis | Infections and infestations | 1 (5%) | 0 (0%) | 0 (0%) | 0 (0%) |  |
| Hypnagogic hallucination | Nervous system disorders | 1 (5%) | 0 (0%) | 0 (0%) | 0 (0%) |  |
| Influenza | Infections and infestations | 1 (5%) | 0 (0%) | 0 (0%) | 0 (0%) |  |
| Lyme disease flare | Infections and infestations | 1 (5%) | 0 (0%) | 0 (0%) | 0 (0%) |  |
| Nausea | Gastrointestinal disorders | 1 (5%) | 1 (5%) | 0 (0%) | 1 (5%) |  |
| Norovirus infection | Infections and infestations | 1 (5%) | 0 (0%) | 0 (0%) | 0 (0%) |  |
| Suicidal ideation ^e^ | Psychiatric disorders | 1 (5%) | 0 (0%) | 0 (0%) | 0 (0%) |  |

| **Note:** Supplementary Table 1 lists frequency and percentage of participants experiencing adverse events for all adverse events occurring during the 6-week study period following the first administration of psilocybin. Adverse events are classified by MedDRA Preferred Term (PT) and System Organ Class (SOC). Incidence of AEs that occurred on the session days are noted here. Incidence of AEs deemed definitely or probably related to psilocybin is also noted here. | | | | | | | |
| --- | --- | --- | --- | --- | --- | --- | --- |
|  | | | | | | | |
| ^a^ For the purposes of adverse event reporting, the 6-week trial period refers to the time from the first psilocybin administration in week 4 of the intervention through the 1-month follow-up in approximately week 10. | | | | | | | |
| ^b^ Relationship of adverse event to the therapeutic intervention was determined by the study team based on temporal proximity, participant attribution, and clinical judgment. Events deemed “probably” or “definitely” related were counted here. | | | | | | | |
| ^c^ The protocol called for the second session to be at 15mg or 25mg depending on response to the first session. In this study 2 participants received a 15mg dose in session 2, while the remaining 18 received 25mg.  ^d^ Pain here included pain in extremities (e.g., arms, legs), neck, back, joints, and general aches.  ^e^ Because of the potentially life-threatening nature of this AE, it was coded as a serious adverse event (SAE). | | | | | | | |
| **Supplementary Table 2.** **Adverse Events (AEs) reported during the 3 to 6-month follow-up period.** | | | | |  |  |  |
| **Adverse Event MedDRA Preferred Term (PT)** | **System Organ Class (SOC)** | **Related, ^a^ n (%)** | **Incidence at 3-mo. follow-up, n (%)** | **Incidence at 6-mo. follow-up, n (%)** |  |  |  |
|  |  |  |  |  |  |  |  |
| Anxiety | Psychiatric disorders | 0 (0%) | 1 (5%) | 1 (5%) |  |  |  |
| Hypnagogic hallucination | Nervous system disorders | 0 (0%) | 1 (5%) | 1 (5%) |  |  |  |
| Depression | Psychiatric disorders | 0 (0%) | 1 (5%) | 0 (0%) |  |  |  |
| Stress | Psychiatric disorders | 0 (0%) | 1 (5%) | 0 (0%) |  |  |  |
| Sleep disturbance | Sleep disorders | 0 (0%) | 1 (5%) | 0 (0%) |  |  |  |
| Diabetes mellitus | Endocrine, metabolic, nutrition disorders | 0 (0%) | 1 (5%) | 0 (0%) |  |  |  |
| Mohs micrographic surgery | Surgical and medical procedures | 0 (0%) | 1 (5%) | 0 (0%) |  |  |  |
| Viral infection | Infections and infestations | 0 (0%) | 0 (0%) | 1 (5%) |  |  |  |
| COVID-19 | Infections and infestations | 0 (0%) | 0 (0%) | 1 (5%) |  |  |  |
| Nausea | Gastrointestinal disorders | 0 (0%) | 0 (0%) | 1 (5%) |  |  |  |
| Fatigue | General disorders and administration | 0 (0%) | 0 (0%) | 1 (5%) |  |  |  |
| Mood swings | Psychiatric disorders | 0 (0%) | 0 (0%) | 1 (5%) |  |  |  |
| Rectal carcinoma, stage 3 ^b^ | Neoplasms | 0 (0%) | 0 (0%) | 1 (5%) |  |  |  |
| Multiple joint pain ^c^ | Musculoskeletal and connective tissue disorders | 0 (0%) | 0 (0%) | 1 (5%) |  |  |  |
| Sprain (wrist) | Musculoskeletal and connective tissue disorders | 0 (0%) | 0 (0%) | 1 (5%) |  |  |  |

| **Note:** Supplementary Table 2 lists frequency and percentage of participants experiencing adverse events for all adverse events reported during the 3 to 6-month follow-up visits following the end of the 6-week study treatment period. Adverse events are classified by MedDRA Preferred Term (PT) and System Organ Class (SOC). Incidence of AEs that were reported at each long-term follow-up are noted here. Incidence of AEs that were deemed definitely or probably related to psilocybin is also noted here. |
| --- |
| ^a^ Relationship of adverse event to the therapeutic intervention was determined by the study team based on temporal proximity, participant attribution, and clinical judgment. Events deemed “probably” or “definitely” related were counted here. |
| ^b^ Because of the potentially life-threatening nature of this AE, it was coded as a serious adverse event (SAE).  ^c^ Multiple joint pain here included pain in knees, shoulders, and back. |

| **Supplementary Table 3. Estimated changes in clinical outcomes at 2 weeks, 1 month, 3 months, and 6 months post-treatment after multivariate outlier removal** | | | | | | |
| --- | --- | --- | --- | --- | --- | --- |
| **Outcome** | **Outliers (N)** | **Time** | **Estimate (95% CI) ^a^** | **% Δ (Baseline)** | **P-value ^b^** | **d_z_ (95% CI) ^c^** |
| **GSQ-30** | 0 | 2 wk | -18.90 (-23.56, -14.24) | -51.7% | < 0.001 | -1.51 (-2.12, -1.20) |
|  |  | 1 mo | -15.45 (-20.11, -10.79) | -42.3% | < 0.001 | -1.12 (-1.71, -0.80) |
|  |  | 3 mo | -16.35 (-21.01, -11.69) | -44.7% | < 0.001 | -1.40 (-2.08, -1.04) |
|  |  | 6 mo | -14.50 (-19.16, -9.84) | -39.7% | < 0.001 | -1.22 (-2.12, -0.78) |
|  |  |  |  |  |  |  |
| **SF-36** |  |  |  |  |  |  |
| *Mental  Summary  Score* | 0 | 2 wk | 6.85 (2.96, 10.75) | 17.2% | < 0.001 | 0.69 (0.32, 1.17) |
|  |  | 1 mo | 6.37 (2.47, 10.26) | 16.0% | 0.002 | 0.64 (0.24, 1.15) |
|  |  | 3 mo | 7.14 (3.24, 11.03) | 18.0% | < 0.001 | 0.73 (0.37, 1.18) |
|  |  | 6 mo | 4.98 (1.08, 8.87) | 12.5% | 0.013 | 0.46 (0.05, 0.93) |
|  |  |  |  |  |  |  |
| *Physical Summary  Score* | 1 | 2 wk | 5.67 (2.57, 8.77) | 14.4% | 0.001 | 0.89 (0.60, 1.33) |
|  |  | 1 mo | 6.45 (3.35, 9.54) | 16.4% | < 0.001 | 0.93 (0.62, 1.38) |
|  |  | 3 mo | 6.95 (3.85, 10.04) | 17.7% | < 0.001 | 0.84 (0.32, 1.82) |
|  |  | 6 mo | 5.78 (2.68, 8.88) | 14.7% | < 0.001 | 0.64 (0.17, 1.40) |
|  |  |  |  |  |  |  |
| **BDI-II** | 3 | 2 wk | -8.59 (-11.44, -5.73) | -61.3% | < 0.001 | -1.14 (-1.91, -0.71) |
|  |  | 1 mo | -9.12 (-11.97, -6.26) | -65.1% | < 0.001 | -1.11 (-1.74, -0.76) |
|  |  | 3 mo | -9.24 (-12.09, -6.38) | -66.0% | < 0.001 | -1.15 (-1.81, -0.75) |
|  |  | 6 mo | -9.00 (-11.86, -6.14) | -64.3% | < 0.001 | -1.13 (-1.73, -0.80) |
|  |  |  |  |  |  |  |
| **PSQI** | 0 | 2 wk | -4.70 (-6.19, -3.21) | -39.2% | < 0.001 | -1.11 (-1.57, -0.82) |
|  |  | 1 mo | -3.90 (-5.39, -2.41) | -32.5% | < 0.001 | -0.92 (-1.35, -0.65) |
|  |  | 3 mo | -5.95 (-7.44, -4.46) | -49.6% | < 0.001 | -1.18 (-1.68, -0.87) |
|  |  | 6 mo | -5.50 (-6.99, -4.01) | -45.8% | < 0.001 | -1.17 (-1.73, -0.85) |
|  |  |  |  |  |  |  |
| **FSS** | 1 | 2 wk | -1.65 (-2.22, -1.08) | -32.1% | < 0.001 | -0.98 (-1.38, -0.75) |
|  |  | 1 mo | -1.53 (-2.10, -0.96) | -29.8% | < 0.001 | -0.90 (-1.24, -0.68) |
|  |  | 3 mo | -1.35 (-1.92, -0.78) | -26.3% | < 0.001 | -0.87 (-1.61, -0.46) |
|  |  | 6 mo | -1.32 (-1.89, -0.75) | -25.7% | < 0.001 | -0.88 (-1.43, -0.46) |
|  |  |  |  |  |  |  |
| **SF-MPQ** |  |  |  |  |  |  |
| *Sensory*  *Pain  Score* | 2 | 2 wk | -2.83 (-3.93, -1.74) | -54.3% | < 0.001 | -0.88 (-1.68, -0.44) |
|  |  | 1 mo | -2.61 (-3.71, -1.52) | -50.0% | < 0.001 | -0.83 (-1.36, -0.48) |
|  |  | 3 mo | -2.28 (-3.37, -1.18) | -43.6% | < 0.001 | -0.77 (-1.47, -0.37) |
|  |  | 6 mo | -3.00 (-4.09, -1.91) | -57.4% | < 0.001 | -0.96 (-1.58, -0.59) |
|  |  |  |  |  |  |  |
| *Affective Pain Score* | 3 | 2 wk | -1.24 (-1.75, -0.72) | -61.8% | < 0.001 | -0.83 (-1.36, -0.47) |
|  |  | 1 mo | -1.29 (-1.81, -0.78) | -64.7% | < 0.001 | -0.95 (-1.63, -0.56) |
|  |  | 3 mo | -1.29 (-1.81, -0.78) | -64.7% | < 0.001 | -0.95 (-1.43, -0.67) |
|  |  | 6 mo | -1.00 (-1.51, -0.49) | -50.0% | < 0.001 | -0.69 (-1.19, -0.30) |
|  |  |  |  |  |  |  |
| *Total  Pain  Score* | 2 | 2 wk | -4.06 (-5.52, -2.59) | -55.7% | < 0.001 | -0.96 (-1.65, -0.58) |
|  |  | 1 mo | -4.39 (-5.85, -2.93) | -60.3% | < 0.001 | -1.16 (-2.02, -0.76) |
|  |  | 3 mo | -3.22 (-4.68, -1.76) | -44.3% | < 0.001 | -0.96 (-1.79, -0.49) |
|  |  | 6 mo | -4.39 (-5.85, -2.93) | -60.3% | < 0.001 | -1.05 (-1.87, -0.63) |

**Note.** Values reflect estimated mean change from baseline derived from linear mixed-effects models fitted by restricted maximum likelihood (REML) with a random intercept for participant. Time (baseline, 2 wk, 1 mo, 3 mo, 6 mo) was modeled as a categorical fixed effect.

^a^ 95% CIs for fixed effects were obtained using the Kenward–Roger approximation for denominator degrees of freedom.

^b^ P-values were adjusted using the Benjamini–Hochberg false discovery rate procedure across all baseline–follow-up contrasts (36 total).

^c^ Cohen’s d_z_ = standardized within-subject effect size with percentile-bootstrap 95% CIs (2,000 resamples).

Higher SF-36 scores indicate improved health-related quality of life, whereas lower scores indicate improvement for all other measures.

Abbreviations: GSQ-30 = General Symptom Questionnaire-30; SF-36 = 36-Item Short-Form Health Survey; BDI-II = Beck Depression Inventory-II; PSQI = Pittsburgh Sleep Quality Index; FSS = Fatigue Severity Scale; SF-MPQ = Short-Form McGill Pain Questionnaire.

| **Supplementary Table 3.1. Changes in general Lyme Disease symptom burden and quality of life scores measured at baseline, then at 2 weeks, 1 month, 3 months, and 6 months following the second psilocybin administration.** | | | | | |
| --- | --- | --- | --- | --- | --- |
|  |  |  | **Full sample** |  | **Cleaned sample** |
| **Outcome** | **Parameter** |  | **Estimate (95% CI)** |  | **Estimate (95% CI)** |
| **GSQ-30** | *Fixed effects* |  |  |  |  |
|  | Baseline (Intercept) |  | 36.55 (28.99, 44.11) |  | — |
|  | 2 wk |  | -18.9 (-23.56, -14.24) |  | — |
|  | 1 mo |  | -15.45 (-20.11, -10.79) |  | — |
|  | 3 mo |  | -16.35 (-21.01, -11.69) |  | — |
|  | 6 mo |  | -14.5 (-19.16, -9.84) |  | — |
|  | *Random effects* |  | *SD* |  |  |
|  | Participant (ID) |  | 14.71 |  | — |
|  | Residual |  | 7.40 |  | — |
|  |  |  |  |  |  |
| **SF-36:** | *Fixed effects* |  |  |  |  |
| *Mental  Component  Summary* | Baseline (Intercept) |  | 39.73 (34.32, 45.14) |  | — |
|  | 2 wk |  | 6.85 (2.96, 10.75) |  | — |
|  | 1 mo |  | 6.37 (2.47, 10.26) |  | — |
|  | 3 mo |  | 7.14 (3.24, 11.03) |  | — |
|  | 6 mo |  | 4.98 (1.08, 8.87) |  | — |
|  | *Random effects* |  | *SD* |  |  |
|  | Participant (ID) |  | 10.11 |  | — |
|  | Residual |  | 6.18 |  | — |
|  |  |  |  |  |  |
| **SF-36:** | *Fixed effects* |  |  |  |  |
| *Physical*  *Component*  *Summary* | Baseline (Intercept) |  | 40.12 (35.29, 44.96) |  | 39.25 (34.30, 44.20) |
|  | 2 wk |  | 5.38 (2.14, 8.62) |  | 5.67 (2.57, 8.77) |
|  | 1 mo |  | 5.17 (1.93, 8.41) |  | 6.45 (3.35, 9.54) |
|  | 3 mo |  | 6.67 (3.43, 9.90) |  | 6.95 (3.85, 10.04) |
|  | 6 mo |  | 5.29 (2.06, 8.53) |  | 5.78 (2.68, 8.88) |
|  | *Random effects* |  | SD |  | SD |
|  | Participant (ID) |  | 9.22 |  | 9.33 |
|  | Residual |  | 5.14 |  | 4.79 |

**Note.** Estimates (*b*) reflect mean change from baseline, derived from linear mixed-effects models with a random intercept for participant. Outcome abbreviations: GSQ-30 = General Symptom Questionnaire-30; SF-36 = 36-Item Short-Form Health Survey. One multivariate outlier was removed from the SF-36 Physical Component Summary using Mahalanobis distance.

| **Supplementary Table 3.2. Changes in depressive symptoms, sleep problems, and fatigue measured at baseline, then at 2 weeks, 1 month, 3 months, and 6 months following the second psilocybin administration.** | | | | | |
| --- | --- | --- | --- | --- | --- |
|  |  |  | **Full sample** |  | **Cleaned sample** |
| **Outcome** | **Parameter** |  | **Estimate (95% CI)** |  | **Estimate (95% CI)** |
| **BDI-II** | *Fixed effects* |  |  |  |  |
|  | Baseline (Intercept) |  | 15.30 (10.89, 19.71) |  | 14.0 (10.01, 17.99) |
|  | 2 weeks |  | -8.40 (-11.48, -5.32) |  | -8.59 (-11.44, -5.73) |
|  | 1 month |  | -8.20 (-11.28, -5.12) |  | -9.12 (-11.97, -6.26) |
|  | 3 months |  | -8.85 (-11.93, -5.77) |  | -9.24 (-12.09, -6.38) |
|  | 6 months |  | -9.35 (-12.43, -6.27) |  | -9.00 (-11.86, -6.14) |
|  | *Random effects* |  | SD |  | SD |
|  | Participant (ID) |  | 8.32 |  | 6.82 |
|  | Residual |  | 4.88 |  | 4.17 |
|  |  |  |  |  |  |
| **PSQI** | *Fixed effects* |  |  |  |  |
|  | Baseline (Intercept) |  | 12.00 (9.99, 14.01) |  | — |
|  | 2 weeks |  | -4.70 (-6.19, -3.21) |  | — |
|  | 1 month |  | -3.90 (-5.39, -2.41) |  | — |
|  | 3 months |  | -5.95 (-7.44, -4.46) |  | — |
|  | 6 months |  | -5.50 (-6.99, -4.01) |  | — |
|  | *Random effects* |  | SD |  |  |
|  | Participant (ID) |  | 3.71 |  | — |
|  | Residual |  | 2.37 |  | — |
|  |  |  |  |  |  |
| **FSS** | *Fixed effects* |  |  |  |  |
|  | Baseline (Intercept) |  | 4.93 (4.05, 5.82) |  | 5.14 (4.26, 6.03) |
|  | 2 weeks |  | -1.57 (-2.13, -1.00) |  | -1.65 (-2.22, -1.08) |
|  | 1 month |  | -1.37 (-1.94, -0.81) |  | -1.53 (-2.10, -0.96) |
|  | 3 months |  | -1.28 (-1.85, -0.72) |  | -1.35 (-1.92, -0.78) |
|  | 6 months |  | -1.26 (-1.82, -0.69) |  | -1.32 (-1.89, -0.75) |
|  | *Random effects* |  | SD |  | SD |
|  | Participant (ID) |  | 1.71 |  | 1.66 |
|  | Residual |  | 0.89 |  | 0.88 |

**Note.** Estimates (*b*) reflect mean change from baseline, derived from linear mixed-effects models with a random intercept for participant. Outcome abbreviations: BDI-II = Beck Depression Inventory–II; PSQI = Pittsburg Sleep Quality Index; FSS = Fatigue Severity Scale. Multivariate outliers were removed from the BDI-II (n = 3) and FSS (n = 1) using Mahalanobis distance.

| **Supplementary Table 3.3. Changes in sensory, affective, and total pain scores measured at baseline, then at 2 weeks, 1 month, 3 months, and 6 months following the second psilocybin administration.** | | | | | |
| --- | --- | --- | --- | --- | --- |
|  |  |  | **Full sample** |  | **Cleaned sample** |
| **Outcome** | **Parameter** |  | **Estimate (95% CI)** |  | **Estimate (95% CI)** |
| **SF-MPQ** | *Fixed effects* |  |  |  |  |
| *Sensory*  *Pain*  *Subscale* | Baseline (Intercept) |  | 5.80 (3.93, 7.67) |  | 5.22 (3.68, 6.76) |
|  | 2 weeks |  | -2.60 (-3.85, -1.35) |  | -2.83 (-3.93, -1.74) |
|  | 1 month |  | -2.25 (-3.50, -1.00) |  | -2.61 (-3.71, -1.52) |
|  | 3 months |  | -1.95 (-3.20, -0.70) |  | -2.28 (-3.37, -1.18) |
|  | 6 months |  | -3.10 (-4.35, -1.85) |  | -3.00 (-4.09, -1.91) |
|  | *Random effects* |  | SD |  | SD |
|  | Participant (ID) |  | 3.57 |  | 2.73 |
|  | Residual |  | 1.99 |  | 1.65 |
|  |  |  |  |  |  |
| **SF-MPQ** | *Fixed effects* |  |  |  |  |
| *Affective*  *Pain*  *Subscale* | Baseline (Intercept) |  | 2.50 (1.63, 3.37) |  | 2.00 (1.41, 2.59) |
|  | 2 weeks |  | -1.40 (-2.04, -0.76) |  | -1.24 (-1.75, -0.72) |
|  | 1 month |  | -1.40 (-2.04, -0.76) |  | -1.29 (-1.81, -0.78) |
|  | 3 months |  | -1.10 (-1.74, -0.46) |  | -1.29 (-1.81, -0.78) |
|  | 6 months |  | -1.05 (-1.69, -0.41) |  | -1.00 (-1.51, -0.49) |
|  | *Random effects* |  | SD |  | SD |
|  | Participant (ID) |  | 1.61 |  | 0.93 |
|  | Residual |  | 1.01 |  | 0.75 |
|  |  |  |  |  |  |
| **SF-MPQ** | *Fixed effects* |  |  |  |  |
| *Total*  *Pain*  *Score* | Baseline (Intercept) |  | 8.30 (5.80, 10.80) |  | 7.28 (5.45, 9.11) |
|  | 2 weeks |  | -4.00 (-5.58, -2.42) |  | -4.06 (-5.52, -2.59) |
|  | 1 month |  | -3.65 (-5.23, -2.07) |  | -4.39 (-5.85, -2.93) |
|  | 3 months |  | -3.05 (-4.63, -1.47) |  | -3.22 (-4.68, -1.76) |
|  | 6 months |  | -4.15 (-5.73, -2.57) |  | -4.39 (-5.85, -2.93) |
|  | *Random effects* |  | SD |  | SD |
|  | Participant (ID) |  | 4.84 |  | 3.1 |
|  | Residual |  | 2.51 |  | 2.2 |

**Note.** Estimates reflect mean change from baseline, derived from linear mixed-effects models with a random intercept for participant. Outcome abbreviations: SF-MPQ = Short-Form McGill Pain Questionnaire. Multivariate outliers were removed from Sensory (n = 2) and Affective (n = 3) subscales, and the Total (n = 2) score using Mahalanobis distance.

**Supplementary Figure. Primary and secondary outcomes across baseline and follow-up timepoints.**

These plots display individual scores, distributions, and summary statistics at baseline, 2 weeks, 1 month, 3 months, and 6 months after the second psilocybin session. Larger circles represent the estimated sample mean, with vertical bars indicating 95% confidence intervals derived from the linear mixed-effects model (Kenward-Roger). Triangles represent participants who repeated the 15 mg dose (*n* = 2). All pairwise comparisons differed significantly from baseline at FDR-adjusted p ≤ 0.013.

**Row 1 (primary outcomes):** General Symptom Questionnaire-30 (GSQ-30; higher scores indicate greater general symptom burden), SF-36 Mental Component Summary, and SF-36 Physical Component Summary scores (higher scores reflect better mental and physical quality of life, respectively).

**Row 2 (secondary outcomes):** Beck Depression Inventory-II (BDI-II; higher scores indicate greater depressive symptoms), Pittsburgh Sleep Quality Index (PSQI; higher scores indicate greater sleep disturbance), and Fatigue Severity Scale (FSS; higher scores indicate greater fatigue).

**Row 3 (secondary outcomes):** Short-Form McGill Pain Questionnaire (SF-MPQ) Sensory and Affective subscales and Total score (higher scores indicate greater pain).
