# Supplementary material for: Pilot study of psilocybin in patients with post-treatment lyme disease
Source: Sci Rep. 2026 Feb 25;16:7497. doi: 10.1038/s41598-026-38091-9 (PMC12936178; doi:10.1038/s41598-026-38091-9)
Supplement: Supplementary file 2 — Supplementary Material 2 [file 41598_2026_38091_MOESM2_ESM.pdf]

EFFECTS OF PSILOCYBIN IN VOLUNTEERS WITH PTLD

Study Treatment Manual

____
Volunteer ID

Meeting Type	Date of Meeting
Visit 1 - Week 1	
Visit 2 - Week 2	
△Visit 3 - Week 3	
***Visit 4 - Session 1 - Week 4**	
Visit 4A - Integration 1 - Week 4	
Visit 5 - Week 5	
***Visit 6 - Session 2 - Week 6**	
Visit 6A - Integration 2 - Week 6	
Visit 7 - Week 7	
*Visit 8 - Week 8	
△Visit 9 (LTFU 1) - Week 10	
△Visit 10 (LTFU 2) - Week 18	
*Visit 11 (LTFU 3) - Week 30	

*In-person visit, △ Optional in-person

2203: Effects of Psilocybin in Post-Treatment Lyme Disease
Investigators: Garcia-Romeu, Aucott

Content of Meetings

☐ VISIT 1 - PREPARATORY SESSION 1

AEs / C-SSRS
Schedule Sessions
Overall approach / expectations
Discussing PTLD History / Symptoms
General life discussion

☐ VISIT 2 - PREPARATORY SESSION 2

AEs / C-SSRS
General life discussion #2
Discussing PTLD History / Symptoms
Photo request

☐ VISIT 3 - PREPARATORY SESSION 3

AEs / C-SSRS
Specific Psilocybin Preparation / Intention setting
General life discussion #3

☐ VISIT 4 - PSILOCYBIN SESSION 1

☐ VISIT 4A - NEXT-DAY INTEGRATION OF SESSION 1

AEs / C-SSRS
Read and discuss participant narrative
Discuss how the session may have related to diagnosis / symptoms

☐ VISIT 5 - INTEGRATION SESSION A WEEK AFTER SESSION 1

AEs / C-SSRS
Discuss previous session
Discuss next session

☐ VISIT 6 - PSILOCYBIN SESSION 2

☐ VISIT 6A - NEXT-DAY INTEGRATION OF SESSION 2

AEs / C-SSRS
Read and discuss participant narrative
Discuss how the session may have related to diagnosis / symptoms

☐ VISIT 7 - INTEGRATION SESSION 1 WEEK AFTER SESSION 2

AEs / C-SSRS
Discussion of sessions and symptoms
Discuss upcoming termination

☐ VISIT 8 - FINAL INTEGRATION SESSION 2 WEEKS AFTER SESSION 2

AEs / C-SSRS
Blood Draw
Discussion of sessions and symptoms

☐ VISIT 9- LTFU 1 (WEEK 10/ 1 MONTH)

AEs / C-SSRS
Discuss progress and symptoms

☐ VISIT 10- LTFU 2 (WEEK 18/ 3 MONTHS)

AEs / C-SSRS
Discuss progress and symptoms

☐ VISIT 11- LTFU 3 (WEEK 30/ 6 MONTHS)

AEs / C-SSRS
Blood Draw
Discuss progress and symptoms

Visit 1 - Week 1

Total Time: 1.5 - 2 hours

☐ OVERALL APPROACH

The general approach of this program is to provide a safe and supportive space to explore the impact of two psilocybin sessions on your physical and mental health. The program is carefully designed for these two sessions to take place in a psychologically supportive environment that is comfortable and allows you to have an introspective experience.

Psilocybin sessions: *The altered state of consciousness provided by psilocybin may allow you to view yourself from a radically broader and deeper perspective. This can provide an opportunity for deep reflection about your life. We hope these sessions will complement the supportive environment and allow you to gain useful insights into your health and wellbeing.*

It is important to remember that this intervention has little to do with the recreational use of psilocybin. We believe that carefully conducted psilocybin sessions can help improve people's health and wellbeing. We would not necessarily expect the same effect from the casual or recreational use of psilocybin.

Unlike most medications, the effectiveness of psilocybin is not thought to be a purely biological effect. In contrast, psilocybin has the ability to profoundly alter your awareness, which, in the right setting and with appropriate intention, may provide you with a broad-based view of your own life and mind, and useful insights.

The psilocybin sessions may do any or all of the following:

- *Often in therapeutic psilocybin sessions, people have experiences in which they feel an incredible sense of agency or creative force.*
- *People tend to remember these experiences, so they may help to provide 'big picture' perspectives long after the sessions.*
- *The sessions can help in coping with daily stress you experience.*
- *Research indicates that the profound experiences that happen in therapeutic psilocybin sessions could have a lasting effect in reducing anxiety and depression.*
- *Therapeutic psilocybin sessions can help people see their lives from a different vantage point.*
- *Situations previously seen as problems and burdens may be seen as opportunities to learn and overcome life challenges.*
- *The sessions can help you change how you prioritize values in life.*
- *After a therapeutic psilocybin session, some people report changing priorities, such as valuing things like one's health, relationships, art, and meaning in life more, while placing less value on things like temporary pleasures, material possessions, and money.*

- *The sessions can help you to change the way you orient yourself concerning the future, such that you now act in your long-term holistic benefit, rather than acting in response to immediate desires.*

- *In therapeutic psilocybin sessions, some people report profound experiences that change their view of the nature of time.*

- *Some people report that they can view their life as a whole across time, as if their life is an unfolding story embedded with meaning.*

- *The sessions can increase your awareness about your automatic behaviors and therefore empower you to shift to intentional, voluntary behaviors.*

The current study will use two doses of psilocybin about 2 weeks apart. The first dosing session will happen in about a month and will use a moderate dose of 15mg. You should be able to feel changes in your thinking, perceptions, and emotions, but these should not be very intense. The idea is to start with a gentle experience to let you get familiar with the drug effects and explore how this might impact your physical and mental health. If all goes well and you're willing, we will go onto a higher 25mg dose about 2 weeks later, which should have stronger effects. This is the dose that has been commonly used in trials for depression and showed good effects in improving mood up to several months later. Psilocybin has not been tested in people with PTLD before, so we cannot know how this will interact with your condition, but based on previous research there is good reason to believe psilocybin could be helpful in reducing problems with mood, fatigue, and quality of life. However, we cannot promise any such effects and will be tracking your mood and health over time to see if psilocybin in this supportive setting provides any short or long-term benefits. Do you have any questions about psilocybin or the study at the moment?

Have you had previous experience taking psilocybin or similar psychedelics like LSD, ayahuasca, mescaline, MDMA, ketamine, salvia divinorum, DMT, etc.? (If yes, ask more about the timing and nature of those experiences. If not, answer any immediate questions, and explain we will discuss the drug effects in more detail later in the study treatment.)

Notes:____
____
____
____
____
____

☐ SCHEDULE PSILOCYBIN SESSIONS

Refer to guide and participant calendars for weeks 4 and 6 and try to determine best days to hold the first and second psilocybin sessions and note those down here. Make sure someone will be available to pick up the participant. Session Date 1:_________________ Session Date 2:_________________

To get the ball rolling on our preparation for your upcoming sessions, it's helpful for us to get to know you. We will typically spend a good amount of time in each meeting learning more about your life history, and important people, places, and events in your life. We'll ask about certain key areas like your early family life and childhood upbringing, your time in school, your friendships and romantic relationships, your education and career, your experience with PTLD, and your general

worldview. This will help us track not only the important formative events from your life, but also to understand the types of things that could come up during your psilocybin experiences. For that reason, it's best for you to be as honest and open as you feel comfortable with, and not to shy away from anything that you feel is taboo or embarrassing. We are open to talking about anything in a compassionate and nonjudgmental way, and want to foster an environment of openness throughout the entire process. Everything you say here is confidential, meaning we will not share this information with any outside parties, with just a few rare exceptions, specifically if you tell us you are planning to harm yourself or others, or have engaged in any harmful or abusive acts towards others we may have to report that out of concern for people's safety. Otherwise, everything said here stays between us. Also, if there's anything you'd like to know about us, don't hesitate to ask.

☐ General Life Discussion

There are 5 major areas that should be addressed over the first three sessions. The length of time spent on these topics will be flexible depending on the participant, but all 5 topics should eventually be addressed over the first three sessions. **You should at least address 2 topics in the first session. Although the primary focus of these discussions will not be PTLD, for each of the 5 topics ask the participant how their diagnosis relates to that domain**.

Childhood and early family life

____
____
____
____
____
____

Current and past relationships including family and friends, past important relationships and events

____
____
____
____
____
____

Romantic life (e.g., current and past partner situation)

____
____
____
____
____
____
____
____

Work and other important activities (interests, hobbies)

____
____
____
____
____
____
____
____
____

World view (e.g., spirituality, religion, metaphysical beliefs, beliefs about death, views about illness, biggest fears/worries)

____
____
____
____
____
____

Topics may be addressed in the order above, although they may also be addressed in a different order as well, depending on the course of conversation and inclinations of the participant. To ensure you will remember what topics need to be addressed in the second session, make sure to mark which topics have been addressed in the first prep session above and keep track for future discussions.

☐ DISCUSSING LYME SYMPTOMS / HISTORY

***FOR FACILITATORS:**
It is important to remember, as a facilitator, that this population can feel invalidated, ignored, and even traumatized by the medical community. Recognize what you are bringing to the conversation, and leave space for the participant to process material. The participant may prefer identity-first language, such as post-treatment Lyme disease participant, or person-first, such as participant with PTLD. Allow the participant to feel autonomous in their preferences and experiences, while still validating what they are telling you. Remember they are the expert.

We'd like to switch gears a little now to talk more about Post-treatment Lyme Disease and get a better understanding of your experience. We'll continue to talk more about this in future meetings, but for now, we like to start off with our very basic understanding of what Post-Treatment Lyme Disease looks like and hear more about how this has impacted your life.

As we understand it, PTLD is considered a third stage of Lyme Disease, after post-localization and dissemination. It seems the progression to Post-treatment Lyme can be multifactorial, in that an individual could develop this diagnosis due to tick-borne infection, immunity dysregulation, chronic inflammation and/or neural network alteration. While there are no currently available treatments, we hope this research could one day be helpful for designing effective therapies to help people with PTLD. It is important to remind oneself that this diagnosis is not the fault of any individual, and what you experience is valid. Should our research not provide the result you hoped for, we can provide an

active list of resources to aid in your aftercare. The Lyme Disease Research Center at Johns Hopkins identifies the following symptoms of PTLD:

- *Fatigue*
- *Musculoskeletal pain*
- *Cognitive difficulties*
- *Sleep disturbances*
- *Decreased quality of life*

Do you currently experience any of these symptoms? If so, which ones are most challenging? Why is that? Do you have other symptoms not listed here? If so, what are those and how do you see those fitting into the picture? Have you found any helpful ways of coping with these issues? If so, what methods have you found helpful and how often are you able to engage in those methods?

Notes:____

Visit 2 - Week 2

Total Time: 1.5 - 2 hours

☐ GENERAL LIFE DISCUSSION # 2

Today, we'll spend some more time discussing your life background and general worldview and how these may relate to your PTLD symptoms.

Make sure to discuss areas that were not discussed from previous sessions, or that need more discussion.

Childhood and early family life

____
____
____
____
____
____

Current and past relationships including family and friends, past important relationships and events

____
____
____
____
____
____

Romantic life (e.g., current and past partner situation)

____
____
____
____
____
____

Work and other important activities (interests, hobbies)

____
____
____
____
____
____

World view (e.g., spirituality, religion, metaphysical beliefs, beliefs about death, views about illness, biggest fears/worries)

____
____
____
____
____
____

Topics may be addressed in the order above, although they may also be addressed in a different order as well, depending on the course of conversation and inclinations of the participant.

PTLD History and Symptoms

____
____
____
____
____
____
____
____
____
____
____
____
____
____
____

☐ PHOTO AND MUSIC REQUEST

You should begin putting together a set of photos for your psilocybin sessions. We will place these around the room on your session days. These can be helpful to make you feel comfortable and emotionally supported during the sessions. You can bring in photos such as:

- *A relatively current photo of yourself*
- *3 or 4 photos of you at different ages in your life. For example: Childhood, teenager, young adult.*
- *Photos of your family members or close friends.*
- *Photos of pets.*

You can also bring any objects that are meaningful like books, dolls, crystals, etc. to make the space feel homier. Additionally, you are invited to start compiling a playlist of music, about an hour's worth should be fine. The easiest way to share this would be through Spotify or to download audio files onto a flash-drive, but we can discuss logistics as needed. The music can be

any songs or pieces you find particularly meaningful or beautiful, that remind you of memorable times in your life, or that you would simply like to listen to and have present during your session days. We will be playing a long standardized playlist throughout most of the session that is largely made up of classical music, but can play your personal playlist if you choose to bring one, usually about mid-day. Do you have any questions or concerns at the moment?

Next week will be the final preparation visit before the first psilocybin dosing session. We will talk more about your life history but also go into detail about what to expect during the psilocybin sessions. How are you feeling about all that at the moment? Do you have any potential goals or intentions in mind at the moment? If not, think about it, and we can discuss further next time.

The next meeting can be held in person or virtually. The in person option can be helpful to get oriented to the space and to lay down on the couch and listen to music with your eyes covered like you will be doing on the session day, but if the travel or your schedule makes this difficult we can meet virtually instead. Which would you prefer?

Is there anything else you'd like to discuss?

Visit 3 - Week 3

Total Time: 1.5 - 2 hours

☐ **GENERAL LIFE DISCUSSION #3**

Now, we'll spend some time discussing each aspect of your life background and general worldview before we dive into some specific preparation for your session. Do you have any questions?

Make sure to discuss areas that were not discussed from previous sessions, or that need more discussion.

Childhood and early family life

____
____
____
____
____
____

Current and past relationships including family and friends, past important relationships and events

____
____
____
____
____
____

Romantic life (e.g., current and past partner situation)

____
____
____
____
____
____

Work and other important activities (interests, hobbies)

____
____
____
____
____
____

World view (e.g., spirituality, religion, metaphysical beliefs, beliefs about death, views about illness, biggest fears/worries)

____
____
____
____
____
____

PTLD History and Symptoms

____
____
____
____
____
____
____
____
____

Setting an intention for session 1

Ask participant if there is any goal or particular intention they have in mind for their first session. What would be most helpful for them in their current state? What would they like to learn or encounter ideally during their session? Remind them that while we can’t control or predict what will happen, it can be useful to set an intention beforehand and we can come back to this after the session.

____
____
____
____
____
____
____
____

☐ Specific Psilocybin Preparation

Prepare for intensive (moderate to high dose) psilocybin session:

Insofar as possible, minimal stress and adequate sleep in days immediately before session. If awake during night before, “think good thoughts.” Don’t worry about insomnia. Not a problem at all. Some techniques require people to stay awake for days to achieve an altered state of consciousness. A sleepless night will not interfere with the psilocybin session.

Breakfast: Be sure to have something light by about 7:00-7:30 AM (e.g., fruits, yogurt, toast, an egg, granola bar, protein shake, etc.). Avoid high fat, heavy foods like sausage, bacon, or biscuits.

Limit caffeine to normal amount. No poppy seeds or grapefruit before sessions. Vitamins and regular medications OK. Do eat something, as empty stomach may be more prone to nausea, and try to eat the same, or similar, breakfast before all sessions. This can affect drug absorption.

Let's figure out what your breakfast plan will be now (record below and have participant write breakfast plan down)

____

____

____

____

No unapproved medications, drugs, alcohol, or herbal supplements the day before the session. Herbal medications need to be evaluated/approved 3 weeks before the session to determine if they could interact with the psilocybin. **Ask about any current or new medications and check with study physician if there are any questions.**

No alcohol 24 hours before and after the session. No driving a car (or operating dangerous machinery) for at least 12 hours after the end of the session.

Arrive by 8:00 am for a urine sample. Comfortable clothing/favorites (nothing too thick on upper arms) so we can take your blood pressure easily.

Session begins around 9:00 am; Baseline BP, after sitting for 5 minutes; Then capsule with water, drink all water in goblet. Briefly examine your reasons to volunteer, and your intention for the session. Time to look at photos, art books, trip to bathroom if needed.

To couch with eyeshade and headphones 15-30 minutes after ingestion; couch made up as bed; flannel sheets/blankets to adjust for temperature changes.

Onset typically between 15-45 minutes: deepening of visual space (blackboard before face vs. night sky); dots of light; muscular relaxation; change of time perception.

Enjoy changes of perception: "Isn't this interesting!" Colors may be more vivid; things in the room might look different or like they are moving; body may feel bigger or smaller; synesthesia (may see sound as colors, etc.). Feel free to explore the emerging experiences.

Basic mantra for the session: Trust, Let Go, Be Open. If feeling anxious, let us know. We can practice mindful breathing, relaxation, simply observing/following your sensations. Let the Music Carry You; Trust the Trajectory (like pathway of a boomerang)— as you go deeper, remember that the effects will eventually wear off, bringing you back to the everyday world.

You may feel like you are dying, melting, dissolving, exploding, going crazy, etc.—go ahead and let yourself surrender into these experiences. Although it may feel very real, like a dream, it is in your mind. Death may symbolize a transcendence of the everyday self, which may be followed by a sense of rebirth and eventually return to the normal world of space & time. Safest way forward is to trust yourself unconditionally. Trust in the wisdom of your own mind; trust in our relationship; if appropriate, trust in any Higher Power/Ground of Being/Source, etc.

We can provide interpersonal grounding: You'll never be alone during the period of drug-action. We can also provide physical support for you during your dosing session. We default to handholding if you are distressed or disoriented, which can sometimes be helpful. If you feel you require more interpersonal support through touch, we may touch your arm or shoulder or pat your back. Any touch beyond that is not allowed between us, and we do not engage in any kind of sexual touch. We consider consent an ongoing process and will clearly ask for consent during your dosing session, especially if we are engaging in any touch like handholding.

Do you consent to this? Are you comfortable with potential physical support during your session?

a. If participant says no, **"We understand. The only times we will breach this agreement is if we need to physically support you for safety reasons (e.g., to make sure you don't run into something or fall down). In those rare instances, we will be sensitive and mindful of any contact and note what is happening out loud, so you are not caught off guard.**

____
____
____
____
____
____

Moving on, we hope you can welcome, confront, dive into, embrace whatever your experience brings. If visual imagery appears, engage: climb staircases, open doors, explore paths, fly over landscapes, etc. However, intense visual imagery may not occur.

Regardless of what you feel or encounter, a general orientation toward engagement rather than retreat tends to be most useful. Directly confront anything potentially frightening; look monster in the eyes and move towards it; "What message do you have for me," or "What can I learn from you?" Look for the darkest corner in the basement and shine your light there.

Challenges may come in many forms. Monster examples before are only one. Challenges may be much more difficult than dealing with frightening images on some sort of "internal movie screen." The challenge may be faceless: blind feelings of fear and terror. You may question why you agreed to do the study and take psilocybin. You may think you are going to be permanently crazy. You may think that you've been overdosed. You may feel that your heart has been pulled out of your chest, or like your soul has been left your body. Or you may feel completely dissociated and confused, as if you don't know what it means to be human anymore. No matter what the circumstance, try to go with the flow and face the terror, confusion, and discomfort. We will support you and keep you safe.

You may be more aware of usual bodily processes: breathing, heartbeat, flow of energy; Trust the parts of your mind that take care of those things in everyday life; You don't have to take responsibility for them just because you are more aware of them.

Basic "default" posture: on back with eyeshade and headphones. Generally, let us adjust them and remove them. Feel free to say, "I'd like to take a break pretty soon;" or "Before long I should go to the bathroom." Usually eyes closed and covered allows for least distractions. Alternate with times of sitting and talking on the couch as needed; exploring altered perceptions in room (which tend to come and go with psilocybin).

Environmental transformations (if they occur) while sitting on couch with open eyes: Take time to "be with" photographs, art on walls. Nothing to fear—perhaps important insights to discover.

You may feel like you need to move your hands or body while on the couch. May be dance-like. May feel like you need to do so to "discharge energy" you feel in your body. This is ok. We will remind you to stay on the couch for your safety.

All is welcome; trust the wisdom of your own mind. These experiences can come in many shapes and sizes and can change from moment to moment. Some people have described parts of their experiences as transcendent, visionary, healing, insightful, or aesthetic —all come as gifts. A lot of different types of experiences and emotions may occur during the day; accept them as they present themselves.

All emotions are welcome; laughter, tears, anger, awe, sexual feelings; be yourself, open & honest. Some people have experiences that replay memories from their lives. Others have experiences that focus on important relationships. Others have described experiences they consider spiritual or 'cosmic.' Whatever type of experience you have, we want to accept it and try to understand what it could mean or provide in terms of helpful takeaways for your life later, but this is something we will do afterwards. During the experience, we just want you to be fully present and absorb whatever happens, and we will come back to talk about the experiences later. You can share as much or as little as you feel comfortable sharing with us.

Role of Intellect: Take the day off; let it play outside; possibility of many new ideas to think about on return. Intuition, not "thinking" during the period of psilocybin-action. Embrace paradoxicality; some parts of the experience may simply be beyond language.

About Music: Just let it wash over you; don't have to like it but try to accept it. What is irritating one moment may become beautiful and inspiring the next. We use a standardized program. We may pause for silence now and then or turn it up or down to make sure you are comfortable. Use headphones when possible (speakers will be on in the background).

Video recording; remember it is basically the behavior of your guide that's being recorded for safety and research purposes. This is nothing to feel embarrassed or concerned about. All recordings are fully confidential, meaning they are not accessible outside our study team. If you have any questions or concerns about this, let us know.

Food: fruit later in day. Bring sandwich or light lunch if you like.

Your friend or family member should arrive around 4:30pm to pick you up. You should be feeling mostly back to normal about 6 hours after taking the capsule, and at that point we can debrief and may ask you to complete some questionnaires if and when you are ready.

We will be collecting you BP/Pulse Data periodically throughout the day. This is normal and nothing to worry about. You will feel the cuff inflating and deflating on your arm periodically. We'll usually leave it on your arm if possible to minimally disturb you. Your BP varies with emotional arousal, like a roller coaster. If it should remain very high a doctor might come in to administer a medication to lower it, but that will not otherwise affect your experience.

Dealing with nausea; emesis basin if needed; no big deal. Deep breathing may help. "Dive into your stomach." If it doesn't go away, let us know; we'll support you if you need to spit something up, then lie right back down and continue your journey. No apologies necessary.

Loss of bladder control is extremely rare, but no big deal if it should occur. There is a protective sheet on the couch. If we suggest a trip to the bathroom, it's probably time to do so and we can escort you there and back safely. You can return to couch and dive right back into experiences afterwards.

Checking in is an invitation to speak; not a requirement. There may be times of deep relaxation or absorption in the experience when communicating can be difficult. You may feel amid an unfolding experience and not want to interrupt it. In those cases, gestures can be a great help. Give us a thumbs up sign if you are feeling OK. If you are feeling anxious or worried in any way, just stick your hand out like a handshake, and one of us will come to check in on you. We value occasional sharing/feedback, but no need to "entertain us." As a whole, "collect experiences" to discuss and interpret later.

Session notes; if you want us to record something, say so. We periodically fill out a checklist reflecting what's going on and will take notes on what happens during the session for our records.

Summary: All is welcome. Think of the session day as an open house in your mind. There is nothing to avoid. If it comes to you, greet it. Your experience may or may not contain imagery, ideas, or feelings about your PTLD. That's ok. We don't know if the effectiveness of this treatment is dependent on specific PTLD-related content in the session. Realizations may come afterward.

In conclusion, this is your day. There should be no interruptions, and we'll make sure to shut off your phone and any other distractions to allow you to engage fully. Take an attitude of perseverance through whatever unfolds, and remember, if you cross some difficult terrain, we'll support you through it.

As a side note, it has happened that a fire alarm may go off during the session. If that happens, don't worry, one of us will check it out. If we need to, we can go outside the building and wait until the all clear is given to return. This should not cause any problems for your session and we will be sure to keep you safe and informed of what is happening.

Afterwards, plan for a quiet evening and light meal. Your facilitator's cell phone number is provided in case you have any questions or concerns after the session.

That evening or the following morning, plan to get some rest and reflect on your experience. Ideally, we would like you to write a session narrative detailing what you remember from the day, or at least a rough draft before your next follow-up appointment (usually the day after session).

Any questions or concerns?

Have volunteer listen to a piece of music from the session while lying on the couch with eyeshades (optional in-person).

____

____

____

____

____

____

____

____

Visit 4 – Week 4 (Psilocybin Session 1)

Total Time: 8-9 hours

Visit 4A- Week 4 (Integration: 1 Day Post Session 1)

Total Time: 1 hour

☐ **PARTICIPANT NARRATIVE & DISCUSSION**

Discuss the participant's narrative about his/her first session and note any potential aftereffects. Discuss how the session may have related to his/her PTLD diagnosis. This must be directed by the participant's experience so the specifics cannot be known in advance. General guidance is to elaborate and discuss any and all aspects of the session as it relates to their health and wellbeing.

Visit 5 - Week 5

Total Time: 1.5 - 2 hours

☐ **<u>DISCUSSION OF PROGRESS</u>**

Determine the participant's progress and continue to unpack material from first session. Have there been any notable shifts or changes in the participant's health or wellbeing?

☐ INTENTIONS FOR NEXT SESSION

Discuss how they felt about their first session and whether they would like to proceed with a second session. Talk about dosing parameters for sessions 1 vs. 2 (15mg vs. 25mg). If proceeding to another session, ask about any concrete intentions or themes they would like to explore.

Visit 6 – Week 6 (Psilocybin Session 2)

Total Time: 8-9 hours

Visit 6A - Week 6 (Integration: 1 Day Post Session 2)

Total Time: 1 hour

☐ PARTICIPANT NARRATIVE & DISCUSSION

Discuss the participant's narrative about his/her second session and note any potential aftereffects. Discuss how the session may have related to his/her PTLD diagnosis and how it compared to the first. This must be directed by the participant's experience so the specifics cannot be known in advance. General guidance is to elaborate and discuss any and all aspects of the sessions as they relate to their health and wellbeing.

Visit 7 – Week 7

Total Time: 1.5 - 2 hours

☐ **DISCUSSION OF PROGRESS**

Determine the participant's progress:

☐ **DISCUSSION OF UPCOMING TERMINATION AND GOALS FOR FUTURE**

Talk about the course of the study treatment coming to an end. How does the participant feel about that? Have they felt like they have gotten anything valuable from their experience so far? If not, what are ways they can make the most of the following weeks? What, if any, are the long-term goals to focus on? Are there any barriers or concerns that can be anticipated going forward and how should those be managed? Would a clinical referral for aftercare be useful or appropriate to explore?

Visit 8 - Week 8

Total Time: 1.5 - 2 hours

☐ **DISCUSSION OF PROGRESS**

Determine the participant's progress and review their overall study experience. Make sure to schedule upcoming follow-up visits.

Visit 9 - 1 Month LTFU

Total Time: 1-2 hours

Have a general discussion with the participant about how they are feeling, hurdles, successes, and other details of their life and study experience. What do they remember most from their experiences? Has their study experience had any lasting impact? Make sure to schedule upcoming follow-up visits.

Visit 10 - 3 Month LTFU

Total Time: 1-2 hours

Have a general discussion with the participant about how they are feeling, hurdles, successes, and other details of their life and study experience. What do they remember most from their experiences? Has their study experience had any lasting impact? Make sure to schedule final follow-up visit.

Visit 11 - 6 Month LTFU

Total Time: 1-2 hours

Have a general discussion with the participant about how they are feeling, hurdles, successes, and other details of their life and study experience. What do they remember most from their experiences? Has their study experience had any lasting impact? Thank them for their participation and remind them we are here to provide additional support or clinical referrals as needed.
